# Supplementary material for: Assessing species composition and insecticide resistance of Anopheles gambiae complex members in three coastal health districts of Côte d’Ivoire
Source: PLoS One. 2024 Dec 10;19(12):e0297604. doi: 10.1371/journal.pone.0297604 (PMC11630590; doi:10.1371/journal.pone.0297604)
Supplement: Attachment — Submitted filename: PONE-D-24-00912_reviewer.pdf [file pone.0297604.s002.pdf]

PLOS ONE

Assessing complex member composition and insecticide resistance status of Anopheles gambiae s.l. in three coastal health districts of Côte d'Ivoire.

--Manuscript Draft--

**Manuscript Number:**	PONE-D-24-00912
**Article Type:**	Research Article
**Full Title:**	Assessing complex member composition and insecticide resistance status of Anopheles gambiae s.l. in three coastal health districts of Côte d'Ivoire.
**Short Title:**	Anopheles gambiae s.l. assessment in coastal health districts of Côte d'Ivoire
**Corresponding Author:**	kouamé Jackson Koffi Ives<br>Universite Nangui Abrogoua<br>Abidjan, CÔTE D'IVOIRE
**Keywords:**	Anopheles gambiae s.l., pyrethroids resistance, PBO, Chlorfenapyr, clothianidin, Vgsc 995F, Vgsc 995S, Ace-1 280S Coastal Côte d'Ivoire
**Abstract:**	Although malaria is endemic in Coastal Côte d'Ivoire, updated data on the resistance profile of the main vector, Anopheles gambiae sensu lato (s.l.), are still lacking, thus compromising decision-making for an effective vector control intervention. This study investigated the complex members and the insecticide resistance in the Anopheles gambiae s.l. populations in coastal Côte d'Ivoire. Between 2018 and 2020, bioassays were conducted on female An. gambiae s.l. mosquitoes in three coastal health districts (Aboisso, Jacqueville and San Pedro) of Côte d'Ivoire. Pyrethroids deltamethrin, permethrin and alphacypermethrin (1X, 5X and 10X), clothianidin and synergist piperonyl butoxide (PBO) combined with pyrethroid 1X were tested using WHO tube bioassays. Chlorfenapyr was evaluated using CDC bottle bioassays. An. gambiae complex members and Vgsc 995F, Vgsc 995S and Ace-1 280S mutations were identified using polymerase chain reaction (PCR) technique. Overall, Anopheles. gambiae s.l. populations were primarily composed of Anopheles coluzzii (88.24%, n = 312), followed by Anopheles gambiae sensu stricto (s.s.) (7.56%) and hybrids (4.17%). These populations displayed strong resistance to pyrethroids at standard diagnostic doses, with mortality remaining below 98% even at 10X doses, except for alphacypermethrin in Aboisso. Pre-exposure to PBO significantly increased mortality but did not fully restore susceptibility, except for alphacypermethrin in Jacqueville. Clothianidin restored full susceptibility in Jacqueville and San Pedro, while chlorfenapyr restored susceptibility in Aboisso at 100 µg ai/bottle and all three districts at 200 µg ai/bottle. Vgsc 995F mutation dominated, with frequencies varying from 71.2% to 79.3%. Vgsc 995S had low, and showed frequencies ranging from 2.3% to 5.7%. Ace-1 280S prevalence varied between 4.2% and 42.9%. Coastal Côte d'Ivoire's An. gambiae s.l. populations were mainly composed of Anopheles coluzzii and showed high resistance to pyrethroids. Clothianidin, chlorfenapyr, and PBO with pyrethroids increased mortality, indicating their potential use as an alternative for malaria vector control.
**Order of Authors:**	kouamé Jackson Koffi Ives
	Edi Ako Victorien Constant
	Zahouli Bi Zahouli Julien
	Kouamé Adjoua Marie Ruth
	Kacou Kadio Yves Alain
	Yokoly Ndri Firmain
	Gbalegba N'guessan Guy Constant
	David Malone
	Koudou Guibehi Benjamin
**Additional Information:**	

Powered by Editorial Manager® and ProduXion Manager® from Aries Systems Corporation

Question	Response
**Financial Disclosure**<br><br>Enter a financial disclosure statement that describes the sources of funding for the work included in this submission. Review the submission guidelines for detailed requirements. View published research articles from *PLOS ONE* for specific examples.<br><br>This statement is required for submission and **will appear in the published article** if the submission is accepted. Please make sure it is accurate.<br><br>**Funded studies**<br>Enter a statement with the following details:<br>• Initials of the authors who received each award<br>• Grant numbers awarded to each author<br>• The full name of each funder<br>• URL of each funder website<br>• Did the sponsors or funders play any role in the study design, data collection and analysis, decision to publish, or preparation of the manuscript?<br><br>Did you receive funding for this work?	The author(s) received no specific funding for this work.
**Competing Interests**<br><br>Use the instructions below to enter a competing interest statement for this submission. On behalf of all authors, disclose any competing interests that could be perceived to bias this work—acknowledging all financial support and any other relevant financial or non-financial competing interests.<br><br>This statement is required for submission and **will appear in the published article** if the submission is accepted. Please make sure it is accurate and that any funding sources listed in your Funding Information later in the submission form are also	The authors of this article formally declare the complete absence of any conflict of interest among them. Their commitment to integrity and transparency ensures an impartial contribution to the presented research. We declare that there is no conflict of interest regarding the publication of this paper. We certify that we have no financial or personal relationships with individuals or organizations that could inappropriately influence our work, and there is no professional or other personal interest of any nature or kind in any product, service, and/or company that could be construed as influencing the content of this paper.

declared in your Financial Disclosure statement.<br><br>View published research articles from *PLOS ONE* for specific examples.<br><br>**NO authors have competing interests**<br><br>Enter: *The authors have declared that no competing interests exist.*<br><br>**Authors with competing interests**<br><br>Enter competing interest details beginning with this statement:<br><br>*I have read the journal's policy and the authors of this manuscript have the following competing interests: [insert competing interests here]*<br><br>* typeset	
**Ethics Statement**<br><br>Enter an ethics statement for this submission. This statement is required if the study involved:<br><br>• Human participants<br>• Human specimens or tissue<br>• Vertebrate animals or cephalopods<br>• Vertebrate embryos or tissues<br>• Field research<br><br>Write "N/A" if the submission does not require an ethics statement.<br><br>General guidance is provided below. Consult the submission guidelines for detailed instructions. **Make sure that all information entered here is included in the Methods section of the manuscript.**	N/A

**Format for specific study types**<br><br>**Human Subject Research (involving human participants and/or tissue)**<br>• Give the name of the institutional review board or ethics committee that approved the study<br>• Include the approval number and/or a statement indicating approval of this research<br>• Indicate the form of consent obtained (written/oral) or the reason that consent was not obtained (e.g. the data were analyzed anonymously)<br><br>**Animal Research (involving vertebrate animals, embryos or tissues)**<br>• Provide the name of the Institutional Animal Care and Use Committee (IACUC) or other relevant ethics board that reviewed the study protocol, and indicate whether they approved this research or granted a formal waiver of ethical approval<br>• Include an approval number if one was obtained<br>• If the study involved *non-human primates*, add *additional details* about animal welfare and steps taken to ameliorate suffering<br>• If anesthesia, euthanasia, or any kind of animal sacrifice is part of the study, include briefly which substances and/or methods were applied<br><br>**Field Research**<br><br>Include the following details if this study involves the collection of plant, animal, or other materials from a natural setting:<br>• Field permit number<br>• Name of the institution or relevant body that granted permission	
**Data Availability**<br><br>Authors are required to make all data underlying the findings described fully available, without restriction, and from the time of publication. PLOS allows rare exceptions to address legal and ethical concerns. See the PLOS Data Policy and FAQ for detailed information.	No - some restrictions will apply

A Data Availability Statement describing where the data can be found is required at submission. Your answers to this question constitute the Data Availability Statement and **will be published in the article**, if accepted.<br><br>**Important:** Stating 'data available on request from the author' is not sufficient. If your data are only available upon request, select 'No' for the first question and explain your exceptional situation in the text box.<br><br>Do the authors confirm that all data underlying the findings described in their manuscript are fully available without restriction?	
**Describe where the data may be found in full sentences. If you are copying our sample text, replace any instances of XXX with the appropriate details.**<br><br>• If the data are **held or will be held in a public repository**, include URLs, accession numbers or DOIs. If this information will only be available after acceptance, indicate this by ticking the box below. For example: *All XXX files are available from the XXX database (accession number(s) XXX, XXX.).*<br>• If the data are all contained **within the manuscript and/or Supporting Information files**, enter the following: *All relevant data are within the manuscript and its Supporting Information files.*<br>• If neither of these applies but you are able to provide **details of access elsewhere**, with or without limitations, please do so. For example:<br><br>*Data cannot be shared publicly because of [XXX]. Data are available from the XXX Institutional Data Access / Ethics Committee (contact via XXX) for researchers who meet the criteria for access to confidential data.*<br><br>*The data underlying the results presented in the study are available from (include the name of the third party*	All relevant data are within the manuscript.

and contact information or URL).<br>• This text is appropriate if the data are owned by a third party and authors do not have permission to share the data.<br><br>* typeset	
Additional data availability information:	

Powered by Editorial Manager® and ProduXion Manager® from Aries Systems Corporation

Article Click here to access/download;Manuscript;Kouame_et_al.docx

Assessing complex member composition and insecticide resistance status of *Anopheles gambiae s.l.* in three coastal health districts of Côte d'Ivoire

Jackson IK Kouamé[1,2*], Constant VA Edi[2], Julien BZ Zahouli[2,3], Ruth MA Kouamé[2,4], Yves AK Kacou[1,2], Firmain N Yokoly[1,2], Constant GN Gbalegba[5], David Malone[6] Benjamin G Koudou[1,2]

1 Unité de Formation et de Recherche Sciences de la Nature, Université Nangui Abrogoua, Abidjan, Côte d'Ivoire. **2** Environnement et Santé, Centre Suisse de Recherches Scientifiques en Côte d'Ivoire, Abidjan, Côte d'Ivoire. **3** Centre d'Entomologie Médicale et Vétérinaire, Université Alassane Ouattara, Bouaké, Côte d'Ivoire, **4** École Supérieure d'Agronomie, Institut National Polytechnique Félix Houphouët Boigny, Yamoussoukro, Côte d'Ivoire. **5** Unité de lutte antivectorielle, Programme National de Lutte Contre le Paludisme, Abidjan, Côte d'Ivoire. **6** Innovative Vector Control Consortium, Liverpool School of Tropical Medicine, Liverpool, United Kingdom.

* Corresponding author: jackson.kouame@csrs.ci / jacksonkouame2017@gmail.com

Shurt title : *Anopheles gambiae s.l.* assessment in coastal health districts of Côte d'Ivoire

Abstract

Although malaria is endemic in Coastal Côte d’Ivoire, updated data on the resistance profile of the main vector, *Anopheles gambiae sensu lato* (*s.l.*), are still lacking, thus compromising decision-making for an effective vector control intervention. This study investigated the complex members and the insecticide resistance in the *Anopheles gambiae s.l.* populations in coastal Côte d'Ivoire. Between 2018 and 2020, bioassays were conducted on female *An. gambiae s.l.* mosquitoes in three coastal health districts (Aboisso, Jacqueville and San Pedro) of Côte d'Ivoire. Pyrethroids deltamethrin, permethrin and alphacypermethrin (1X, 5X and 10X), clothianidin and synergist piperonyl butoxide (PBO) combined with pyrethroid 1X were tested using WHO tube bioassays. Chlorfenapyr was evaluated using CDC bottle bioassays. *An. gambiae* complex members and *Vgsc* 995F, *Vgsc* 995S and Ace-1 280S mutations were identified using polymerase chain reaction (PCR) technique. Overall, *Anopheles. gambiae s.l.* populations were primarily composed of *Anopheles coluzzii* (88.24%, n = 312), followed by *Anopheles gambiae sensu stricto* (*s.s.*) (7.56%) and hybrids (4.17%). These populations displayed strong resistance to pyrethroids at standard diagnostic doses, with mortality remaining below 98% even at 10X doses, except for alphacypermethrin in Aboisso. Pre-exposure to PBO significantly increased mortality but did not fully restore susceptibility, except

suggest you say “induces ...” instead of “restore”

for alphacypermethrin in Jacqueville. Clothianidin restored full susceptibility in Jacqueville and San Pedro, while chlorfenapyr restored susceptibility in Aboisso at 100 µg ai/bottle and all three districts at 200 µg ai/bottle. *Vgsc* 995F mutation dominated, with frequencies varying from 71.2% to 79.3%. *Vgsc* 995S had low, and showed frequencies ranging from 2.3% to 5.7%. Ace-1 280S prevalence varied between 4.2% and 42.9%. Coastal Côte d'Ivoire's *An. gambiae s.l.* populations were mainly composed of *Anopheles coluzzii* and showed high resistance to pyrethroids. Clothianidin, chlorfenapyr, and PBO with pyrethroids increased mortality, indicating their potential use as an alternative for malaria vector control.

Key Words: *Anopheles gambiae s.l.*, Insecticides resistance, Chlorfenapyr, clothianidin, Coastal Côte d'Ivoire

Keywords: Anopheles gambiae s.l., pyrethroids resistance, PBO, Chlorfenapyr, clothianidin, Vgsc 995F, Vgsc 995S, Ace-1 280S Coastal Côte d'Ivoire

Introduction

The World Health Organization (WHO) has reported recently that there were 249 million cases and 608,000 deaths from malaria in 2022 [1]. Malaria is most prevalent in sub-Saharan Africa, where the majority of cases and deaths occur. Within sub-Saharan Africa, countries such as Nigeria, the Democratic Republic of the Congo, and Mozambique have *to be removed* some of the highest malaria burdens. Malaria is one of the deadliest public health diseases, particularly in sub-Saharan Africa among children less than 5 years old. The WHO African region accounted for 95% of global malaria cases and 96% of deaths [2]. More than US$ 3.5 billion have been invested in 2021 for malaria, with a third of this investment (around US$ 1.1 billion) spent by the governments of malaria-endemic countries [2]. The WHO recommended malaria prevention strategies, including the effective vector control tool that have major impacts in reducing the global burden of this disease. The WHO Global Technical Strategy (GTS) aims to reduce malaria incidence and mortality by at least 75% by 2025 and 90% by 2030 which seems to be challenging [2]. In 2021, 242 million Artemisinin-based Combination Therapies (ACTs) were distributed to the public health sector by National Malaria Control Programs (NMCPs) and about 590 million Insecticide Treated Nets (ITNs) were delivered to communities in the period 2019–2021 [2]. To achieve these goals, GTS has called for the development of new vector control tools that must incorporate new insecticide molecules (e.g., clothianidin and chlorfenapyr), synergists (e.g., pyperonyl butoxide: PBO) and/or insecticide mixtures containing at least two active ingredients with different modes of action to mitigate malaria vector resistance to insecticides [2–4]. Côte d'Ivoire's population lives in high malaria risk areas [5]. In 2021, malaria *No need* burden cases in the country were estimated at 7,443,146 cases and 14,906 deaths [2]. The main objectives of the National Control Malaria Program (NMCP) are to reduce malaria morbidity by 40 percent and by 33 percent the malaria mortality in high-burden by 2026 compared to the 2015 baseline [6]. The vector control strategy implemented by

the NMCP is mainly based on the mass deployment of long-lasting insecticidal nets (LLINs) every three years and recently by implementing indoor residual sprays (IRS) [7–9] in one district. In Côte d'Ivoire, the National Malaria Strategic Plan 2016–2020 has prioritized indoor residual spraying (IRS) as an additional vector control method to reduce malaria morbidity and mortality [10]. The transmission of malaria in Côte d'Ivoire are ensured by *An. gambiae s.l.*, *An. funestus s.l.* and *An. nili* [7,11]. Species from the *An. gambiae s.l.* complex (*An. gambiae s.s.*, *An. coluzzii*, and hybrids) have been identified across the country [7,12–15]. However, Ivorian *An. gambiae s.l.*, populations exhibit strong resistance to many traditional classes of insecticides (i.e., DDT, pyrethroid, carbamate, and organophosphate) [7,13–17]. Local *An. gambiae s.l.* resistance to insecticides is a severe challenge to the NMCP efforts because all

move after PYR

LLINs distributed in Côte d'Ivoire before 2021 were treated only with pyrethroid [7,10].

Should be Strategies in your cases

Therefore, it is urgent to develop effective alternative tools for the sustainable control of insecticide-resistant malaria vectors. So, since 2018, the National Malaria Strategic Plan (NMSP) supported by the U.S. President's Malaria Initiative (PMI) project has contributed to the generation of insecticide resistance data to conduct a stratification of vector control interventions across the country. From 2018 to 2022, the NMCP and PMI deployed IRS in the health districts of Nassian and Sakassou using clothianidin-based insecticides that resulted in

Rephrase sentence from line 89 to 92 , as it may be confusing to reader.

how susceptibility test can be carried out in order to have nets?

effective malaria vector control [6,7]. Insecticide susceptibility tests were also carried out in order to have nets incorporated with pyrethroids and other active ingredients (synergists, pyrroles, etc.) and with dual active ingredients, for the next LLIN distribution campaign, in line with the entomological stratification data [6,7]. Recently, pyrethroids in combination with either an insect growth regulator, a pyrrole, or a synergist that inhibits the primary metabolic mechanism of pyrethroid resistance within mosquitoes were used in LLINs [3,4,7]. Therefore, in the current study, insecticide resistance status and complex members of *An. gambiae s.l.* were assessed in three coastal health districts of Côte d'Ivoire, namely Aboisso, Jacqueville and San

Pedro. The health district of Jacqueville records one of the highest malaria incidences three hundred sixty-seven per mile (367‰), while a lower incidence is reported in the health district of San Pedro (127.1‰) [18,19]. In Côte d’Ivoire, the coastal areas are characterized by a massive concentration of people [20]. These areas are the heart of the economic development of the country and have two main seaports, one in Abidjan and one in San-Pedro. This coastal region is home to the largest number of agro-industrial units, traditional agriculture and smallholdings [11,14,21]. The industrial and traditional crops (e.g., cocoa, rubber, oil palm, coffee, rice) [11,12,14,15,21] are intensively treated with pesticides and insecticides to protect crops [14]. The local *Anopheles* malaria vectors are constantly under strong pressure from insecticides and chemicals, which can lead to the development of their resistance and increased their survival. Therefore, an urgent need is to develop alternative insecticides and evaluate their effectiveness against malaria vectors in resistant areas, mainly on the coastline. The current study is part of a program designed to explore the landscape of malaria *Anopheles* vectors in coastal areas and inform disease control programs of Côte d'Ivoire.

coma after “incidences” on line 97 and suggest change to “thousand” instead

I suggest you say “in Côte d’Ivoire” Instead

Methodology

Study sites

The study was conducted in three health districts, namely Aboisso (latitude 5° 28’ 04” N and longitude 3° 12’ 25” W), Jacqueville (latitude 5° 12’ 02” N and longitude 4° 24’ 44” W) and San Pedro (latitude 4° 44’ 5” N and longitude 6° 38’ 10” W), located in the southern littoral area of Côte d’Ivoire (Fig 1). The local climate is characterized by two major seasons: the long rainy season from April to July, with the peak of rainfalls in June, while the long dry season extends from October to March. The annual rainfall average is 1848 mm with an annual temperature average of 27 °C. In the three health districts, economic activities were dominated

should this be in present tense?
In general, we use the present tense to describe actions and states of being that are still true in the present.

by agriculture leading to large agricultural areas for food crops and cash crops (rubber, oil palm, pineapple, cassava, yam, banana) [20,22,23]. Farmers use several types of pesticides (herbicides, insecticides, and fungicides) to protect crops and increase production [14,21,24].

< Figure 1 near here >

Fig 1: Map of Côte d'Ivoire showing the location of the littoral health district.

Larval sampling and rearing to adults

I suggest you used "different" instead of "diverse"

Surveys for potential mosquito larvae habitats were conducted in diverse locations such as water puddles, vegetable cultivation sites, rice fields, and other potential larval habitats within our study sites. The larvae collected from each health district were combined, regardless of their collection site. *Anopheles gambiae s.l.* larvae were collected from breeding sites (e.g., rice

There is a redundancy here with the prious sentence , considere rephrasing and/or combining them

fields, water wells, natural breeding sites, gardens fields (tomatoes, cabbage, carrot, lettuce))

use "into" instead

from January 2018 to December 2020. Larvae were transferred and reared to adults under standard laboratory conditions (27 ± 2°C temperature; 70 ± 10% relative humidity; 12:12 hour light: dark photoperiod). Emerged adults were provided with a cotton wool pad soaked in a 10% sugar solution. Species were identified morphologically using identification keys [25].

WHO tube bioassay

Consider combining the two sentences from lines 138 to 140,

When were the tests done with the chlorfenapyr then?

The diagnostic dose tests of pyrethroids (alphacypermethrin, deltamethrin, and permethrin) were conducted in 2018, 2019, and 2020. The following tests were conducted exclusively in 2020. These include intensity tests (1X, 5X, and 10X), tests with the synergist (PBO), and clothianidin tests. Emerged adult females of *An. gambiae s.l.* were tested for insecticide susceptibility according to the World Health Organization (WHO) standard procedures [26]. Females of F_0 generations aged 2–5 days were used in all the tests. Four batches of 20–25 non-

in each of the four testing tubes

blood-fed females were introduced each in four tube tests garnished with insecticide-treated filter papers, while two batches were exposed to control tubes with untreated filter papers for

one hour. Mosquitoes were exposed to the WHO discriminating dosages of deltamethrin (0.05%), permethrin (0.75%) and alpha-cypermethrin (0.05%) to determine their resistance status (WHO, 2018). Moreover, synergist effects were assessed by pre-exposing mosquitoes to

Please double check the PBO concentration on the coated paper, should this be 4% insetead

5% PBO for one hour before being exposed to deltamethrin 0.05%, permethrin 0.75% and alphacypermethrin 0.05% for an additional hour. To determine the pyrethroid-resistance intensity, mosquitoes were exposed to 1X, 5X and 10X diagnostic concentrations of alpha-cypermethrin (0.05%, 0.25% and 0.5%), permethrin (0.75%, 3.75% and 7.5%) and deltamethrin (0.05%, 0.25% and 0.5%). Mosquito mortality was recorded at 24 hours after exposure.

For clothianidin, impregnated papers were prepared in *situ* following the standard operating procedure (SOP) developed by the Vector Link entomology team. The diagnostic dose was set

There is need to moved thos down to line 160 before the sentence starting with "Mosquitoes…"

at 2% clothianidin. Clothianidin was tested using WHO susceptibility tests, with the standard guidelines [27]. A solution of clothianidin was prepared by diluting 264 mg of the formulated product (Sumishield WG50) in 20 ml of distilled water. Four Whatman No.1 filter papers of 12 × 15 cm each were impregnated using a pipette to dispense 2 ml of solution on each filter paper, resulting in a concentration of 13.2 mg/ai clothianidin per paper. The untreated filter papers were treated using 2 ml of a solution of distilled water. Mosquitoes were tested against clothianidin-treated papers as described above [28]. The mortality was recorded daily for up to seven days in order to capture any delayed mortality effects.

CDC bottle test

The CDC bottle tests were exclusively conducted in 2020. Chlorfenapyr susceptibility was

Please consider revising the sentence on line 165 starting with "The tests were…" to make it clear to readers

determined using Centers of Disease Control and Prevention (CDC) bottle tests. The tests were

Is there any rational in testing against the diagnostic dose (100µg) and then 200µg (why not other concentration)

used 250 ml glass bottles coated with 100 µg ai/bottle and 200 µg ai/bottle. A set of 15 to 20

should be also two hour starved prior to the testing

non-blood-fed female *An. gambiae s.l.* aged 2–5 days were exposed to two discriminating concentrations of chlorfenapyr following the CDC bottle test protocol [29]. Mortality was

I could not check the protocol in the given ref, so what guided the choice of these number insteaad of using 20 to 25 Mosquitoes

Why 2 days mosquitoes while it is recommend 3-5 old days moqitoes

recorded daily for up to three days.

Please check for the REF 29 on line 168, I am not sure if this is the good reference you should report here, as this refers to information related to epidemiology Do add to this referewnce the link to make it easier to find it online.

rephrase the sentence from line 171, should be "...surviving moqitoes to exposure in WHO tubes....

Molecular analyses of *An. gambiae* s.l. complex members and resistance genes

Dead and surviving mosquitoes' exposure in WHO tube bioassays and CDC bottle tests were separately stored individually in Eppendorf tubes in silica gel and kept at −20 °C for further identification of *An. gambiae* s.l. complex members and *kdr* and *Ace-1* mutations.

General comment:
Please consider removing thoughout the manuscript, the "s.l." or "complex members" as they indicates the same
please revise all the citations in the manuscript as they do not aline with the reference listing

DNA extraction

The genomic DNA of individual mosquito was extracted according to the method described in *Collins et al.* [11]. In the Eppendorf® tube (1.5 ml) individual mosquito was soaked and crushed

Please check this, it does not seem to be correctly referenced, should it be [30]?

in 200 µl of 2% Cetyl Trimethyl Ammonium Bromide (CTAB) and incubated at 65 °C for 5 min. A total of 200 µl of chloroform was added and the resulting mixture was centrifuged for 5 min at 12,000 rpm. The supernatant was pipetted into a new 1.5 ml tube. Then, 200 µl isopropanol was added, mixed by pipetting and centrifuged for 15 min at 12,000 rpm to precipitate the DNA. DNA pellet formed at the bottom of tubes. The supernatant was discarded. With 200 µl of 70% Ethanol purified the DNA of each sample and centrifuged for 5 min at 12,000 rpm. The ethanol was removed, and the pellet dried on the bench overnight. The extracted DNA was reconstituted in 30 µl DNase-free water (Sigma-Aldrich, United Kingdom) and incubated at 65 °C for 15 min and stored in the fridge at -20°C.

the text here seems to be copied from the manufacture guidelines/protocol
Please resentence all these and make it clear what you did instead OR to just refrered to it by citation

Complex member identification

The molecular identification of *An. gambiae* complex members was performed according to the

please check for the right reference

SINE-PCR method [31], with minor modifications of reaction mixture. The PCR assay was performed using two primers, 6.1a (5'-TCGCCTTAGACCTTGCGTTA-3') and 6.1b (5'-CGCTTCAAGAATTCGAGATAC-3'). Each reaction mixture was done in a final volume of 25 µl containing 12.5 µl One taq Quick-load 2X Master Mix, 5.5 µl free water, 0.5 µl Dimethyl sulphoxide (DMSO), 2 µl Bovine Serum Albumin (BSA), 1 µl of each primer, 0.5 µl

Magnesium chloride ($MgCl_2$) and 2 µl DNA template. The incubation took place in a thermocycler of LongGene® type (A200 Gradient Thermal cycler; LongGene Scientific Instruments Co., Ltd Hangzhou, P.R. China) according to the following program: 94 °C for 5 min, 94 °C for 25 sec, and 54 °C for 30 sec; 72 °C for 1 min repeated 35 times; and a final step at 72 °C for 10 min to terminate the reaction. After amplification, PCR products were run on either a 1.5% agarose gel in Tris/borate/EDTA (TBE) and stained with ethidium bromide solution for UV visualization. PCR products were loaded on the gel and allowed to migrate under a voltage of 140 V for one hour. The result was visualized with a UV illuminator (BioDoc- It Imaging System; Upland, CA, USA).

please use "an" instead

Identification of target site mutation

The presence of insecticide resistance genes including *Vgsc*-L995F (previously known as *Vgsc*-L1014F (*Kdr-West*)), *Vgsc*-L995S (previously known as *Vgsc*-L1014S (*Kdr-East*)), and Ace1-G280S (previously known as Ace1-G119S)[32] was investigated using real-time PCR. The TaqMan assays as detailed by Bass [33] were used to screen for the 995F, 995S kdr and 280S mutations. The reaction was carried out in an Agilent Stratagene MX3005 qPCR thermocycler (Agilent Technologies, Santa Clara, CA, USA). For each 1 µl gene DNA, a final volume of 9 µl mix is used. The mix contains DNase-free water (3.875 µl), sensimix (5 µl) and specific primer/Probe (0.125 µl) for *kdr* 995F or 995S or *Ace1*-280S. The specific probe contains FAM and HEX fluorochromes. FAM was used to detect the mutant allele, while HEX detected the wild-type susceptible allele. PCR conditions used were 10 min at 95 °C (1 cycle), followed by 40 cycles of 10 sec at 95 °C, and 45 sec at 60 °C.

Rephrase the sentence from lines 208 - 209, and use "master mix" instead of "mix" alone and do the same for the milar term troughout the manuscript

Data analysis

The resistance status of each mosquito population was determined according to the WHO criteria with mortality after 24-hour, 72-hour and 7-day post-exposure for pyrethroids, chlorfenapyr and clothianidin, respectively [29]. Mortality was corrected using Abbott's formula ($\text{Corrected mortality} = \frac{(\text{Mortality treated} - \text{mortality control})}{(100 - \text{mortality of untreated control})}$X100), when the mortality of the control tubes was above 5% and less than 20% [34]. There was a confirmed resistance if the mortality percentage was <90%, possible resistance if the mortality rate was between 90 and 98%, and susceptibility if the mortality rate was ≥98%. The mortality recorded each year was compared between each insecticide (pyrethroid diagnostic concentration) using the ANOVA test or Kruskal-Wallis. For the resistance intensity, if corrected mortality was 98–100% at 5X the diagnostic dose indicated low resistance intensity; if corrected mortality was less than 98% at 5X diagnostic dose implied testing the 10X diagnostic dose; if corrected mortality was 98–100% at 10X the diagnostic dose confirmed a moderate resistance intensity; if corrected mortality was less than 98% at 10X the diagnostic doses indicated high resistance intensity. For the synergist assays, the mortality of mosquitoes exposed to PBO with pyrethroid was compared with that of the insecticides without PBO pre-exposure. Comparison was made between mortality rates with and without PBO pre-exposure using the *prop .test* with software R version 4.2.1. The frequency of resistance mutations (*Vgsc*-995F, *Vgsc*-995S and *Ace1*-280S) was determined using the formula: F = [(2RR+ RS)] / [2(RR+RS+SS)] [35] with SS, homozygous susceptible genotype; RS, heterozygous genotype; RR, homozygous resistant genotype.

Results

Species composition of the *Anopheles gambiae* complex

Is there a rational of using,
I am not sure why you used ANOVA or xxx to compare proportion data here, as this a transformation before comparison.
Instead I will recommend using the GLM a more modern and right way to analyse binomial data with higher power than analyses of transformed data

Since you did collection from different sites and different years I would suggest doing a GLM and assessing if MR varied between sites according to year of collection by using an interaction between site and year

A total of 672 *An. gambiae s.l.* was selected from the three sites for species identification. Overall, *An. gambiae* complex was mainly composed of *An. coluzzii* (88.2%, n=593), followed by *An. gambiae s.s.* (7.6%, n=51) and hybrids (*An. coluzzii*/*An. gambiae s.s.*) (4.2%, n=27). In Jacqueville and San Pedro, all mosquitoes were identified as *An. coluzzii*. The three forms were found in sympatry in Aboisso. However, *An. coluzzii* dominated in Aboisso (66.7%, n=158), followed by *An. gambiae s.s.* (21.5%, n=51) and finally hybrids (11.8%, n=28) (Fig 2).

On line 240 consider revising terms as An. gambiae ss and An. coluzzii are no longer consider as “forms” bu

< Figure 2 near here>

Revise the legend on the figure, we can really see the line on the pie charte indicating the hybrids proportions

Fig 2: Distribution of members of *Anopheles gambiae s.l.* complex in the three study populations.

In some areas you seem to have high proportion of hybrids how was the mosquitoes stored (individually or in groups per tube) as the later if not managed carefully can allow sharing material such leg between speciment during the DNA extraction

Insecticide susceptibility in *An. gambiae s.l.*

The stats results seem not to be alined with what is seen on the fig 3. Thus, I greatly suggest you consider redoing the analysis according to my suggestion in the data analysis section.

The picture (Fig 3) shows the mortality of *An. gambiae s.l.* populations from Aboisso, Jacqueville and San Pedro against alphacypermethrin, permethrin, and deltamethrin with the WHO diagnostic concentrations in 2018, 2019 and 2020.

Please revise the section on “insecticide susceptibility...” there is no need to recall value for every single site at each year . It seems like a redundancy as you already present this value as a graph (Fig. 3). For example see how you did for the results on the synergist action form the PBO (from line 284)

In 2018, in Aboisso *An. gambiae s.l.* populations, the mortality recorded was 5% for deltamethrin, 6% for permethrin and 0% for alphacypermethrin. The mortalities were not different (df = 2; $p = 0.05$) between insecticides. In 2019, the mortality was 6% for deltamethrin and no mortality was observed for permethrin and alphacypermethrin, the mortality rate recorded were significantly different between insecticides (df = 2; $p = 0.03$). The mortality recorded in 2020 were 2%, 1% and 29% respectively for deltamethrin, permethrin and alphacypermethrin. Between insecticides, the mortalities were not different (df = 2; $p<0.22$).

??? no difference

Please the p value on line 256 , should this be “=“ instead?

In Jacqueville populations, the mortality recorded in 2018 were 5% for deltamethrin, 2% for permethrin and 0% for alphacypermethrin. The mortalities observed were not different (df = 2; $p<0.07$) between insecticides. In 2020, no variation was observed between each insecticide (df

= 2; p = 0.12), the mortality recorded was 11% for deltamethrin, 2% for permethrin and 20% for alphacypermethrin.

In San Pedro populations, in 2018, the mortality rates were 4% for deltamethrin, 2% for permethrin and 0% for alphacypermethrin. The mortality rates recorded in 2019 were 4% for deltamethrin and permethrin and 0% for alphacypermethrin. In 2020, the mortality was 1% for permethrin and no mortality (0%) was observed for deltamethrin and alphacypermethrin. Each year, the mortality rates did not significantly differ between insecticides (Each p>0.05).

< Figure 3 near here >

Fig 3: Susceptibility of *An. gambiae s.l.* to pyrethroids by site from 2018 and 2020. Error bars represent the standard errors at the Y axis and the black dashed line represents the susceptibility threshold

Intensity of resistance

Please be consistant in your writing, consider writing "Fig." (example lines 247 ; 276) or "Figure" but do not mixte them

The results of the pyrethroid intensity test are summarized in Figure 4. These results confirmed the strong resistance of *An. gambiae s.l.* against alpha-cypermethrin, permethrin and deltamethrin. The results of resistance pyrethroid intensity tests, for all three samples from Aboisso, Jacqueville, and San Pedro, the mortalities remained below the 98% threshold when exposed to 1X and 5X concentrations of all insecticides (Fig 4). At 10X concentration, overall high intensity resistance was still observed in all three mosquito populations for the insecticides, except for moderate intensity resistance recorded with alphacypermethrin 0.5% in Aboisso populations with 100% mortality.

lines 274 - 276: the sentence is not very clearly explained, revise the english there

Consider revising the english also the sentence from 276 to 279

< Figure 4 near here >

Could you indicate the number of mosquitoes exposed on the graph instead of the proportion as you did on fig 4

line 281: Please consider using "from" instead of "of"

Fig 4: Intensity bioassays of pyrethroid against *An. gambiae s.l.* of the three study populations. Error bars represent the standard errors and the red dashed line represents the susceptibility threshold

Effect of piperonyl butoxide (PBO)

Table 1 shows the mortality rates in the Aboisso, Jacqueville and San Pedro *An. gambiae s.l.* populations exposed to alpha-cypermethrin, permethrin and deltamethrin without and with pre-exposure to PBO. The results showed that synergist effect of PBO was strongest in all mosquito populations. Mortality increased significantly when mosquitoes were pre-exposed to PBO (df=1, p <0.0001) (Table 1). Although a significant increase was observed in mortality after pre-exposure to PBO, susceptibility was not fully restored. The mortality rates after PBO pre-exposure were still under the 98% threshold in all three study sites, except for the Jacqueville populations in which alphacypermethrin mortality after pre-exposure to PBO was 98.5% (Table 1).

< Table 1 near here >

Table 1: Bioassays of *An. gambiae s.l.* to pyrethroids without and with piperonyl butoxide of the study population. N=represents the total number of mosquitos tested, CI=represents the confidence intervals

Please revise the title on line 295, Bioassays is a methods you cannot have a method within this table thus this cannot be a "bioassay"

In the table 2 and the lines 232, 233 and 340, can you change the letters used in the formula to avoid any confusing for the readers as some of the letters ("SS") you use there are also consider as genotypes (use for example "aa" and "AA")

Effect of clothianidin and chlorfenapyr

Anopheles gambiae s.l. populations from Jacqueville and San Pedro were fully susceptible to clothianidin, with mortalities of 98% (Fig 5). For the San Pedro population, 100% mortality was reached six days after exposure. In the Jacqueville population, the mortality rate was 98.8% seven days after exposure. However, for the Aboisso population, susceptibility was not fully restored after the 7-day post-exposure as the mortality rate was 55.7%.

Sentence from lines 300 to 301, please be more specific here, bars are displaying mortality from different days

lines 306 and 312: is there any rational in using "susceptibility restored" instead of saying the "mortality rate) as "restore here may mean "going back to the original state after a lost..." and here at anytime you mentionned previous resistance to chlorfenapyr.

For chlorfenapyr at 100 µg/bottle, the Aboisso population was fully susceptible, with a 72-hour mortality of 100%. However, chlorfenapyr did not fully restore susceptibility in Jacqueville and San Pedro populations with 100 µg/bottle. With 100 µg/bottle, the 72-hour mortality was of 88.7% for Jacqueville and 97.1% for San Pedro populations (Fig 6). With 200 µg/bottle, the mortality rate was 100% for the San Pedro population after 24-hours post-exposure. Except for the Jacqueville population where 100% mortality was recorded after 48-hours post-exposure (Fig 6). The population of Aboisso was not tested at 200 µg/bottle, because susceptibility was restored at 100 µg/bottle.

????

Please consider removing "of" on line 308

???

< Figure 5 near here >

This is a figure showing results, how can it be a "susceptibility of An. gambiae s.l."

Fig 5: Susceptibility of *An. gambiae s.l.* to clothianidin 2% of the different populations in 2020. Error bars represent the standard errors and the red dashed line represents the susceptibility treshold.

< Figure 6 near here >

Please revise the title on line 318, "CDC bottle bioassays" is a methods you cannot have a method on this figure, thus this cannot be a "CDC bottle bioassay"

Fig 6: CDC bottle bioassay of chlorfenapyr against *An. gambiae s.l.* of the three populations in 2020. Error bars represent the standard errors and the red dashed line represents the susceptibility treshold.

Resistance mutation

Table 2 shows the *kdr* (L995F and L995S) mutation allelic frequencies in the population from Aboisso, Jacqueville and San Pedro (Table 2). The *kdr* L995F mutation allelic frequencies ranged from 79.3% for *An. coluzzii*, 76.0% for hybrids, and to 72.1% for *An. gambiae s.s.* in the Aboisso population. In the population from Jacqueville and San Pedro, the *kdr* L995F (*Kdr west*) frequencies were respectively 38.1% and 71.2%. For the *Kdr* L995S (*Kdr East*), the

Line 325: please use "was" and remove "to" instead

Lines 328- 329: Please consider the following "The mutation allelic frequencies were 2.3% for An. 329 gambiae s.s. in Aboisso population and 5.7% in San Pedro population" instead

mutation allelic frequencies were low. The mutation allelic frequencies were 2.3% for *An. gambiae s.s.* in the Aboisso population and 5.7% for the San Pedro population. For the population of Aboisso (*An. coluzzii*, and Hibryd), and San Pedro mutation was not detected. The mutation allelic frequencies of G280S were summarized in table 2. The mutation allelic frequencies of G280S were 27.4% for *An. coluzzii*, and 42.9% for *An. gambiae s.s.* in the population of Aboisso. The allelic frequencies were 31.1% and 4.2% respectively for the population of Jacqueville and San Pedro.

Lines 330: Please consider "Hybird" instead.

Sentence from lines 328 - 330: Since An. gambiae ss from Aboisso has this mutation , please removed An. coluzzii and Hybrid from the brackets so that it is clear to readres that you are referring to them

< Table 2 near here >

Table 2: *An. gambiae s.l.* sibling species composition and *Vgsc*-995F, *Vgsc*-995S and *Ace-1* 280S frequency in three populations. Hybrids represented *An. coluzzii/ An. gambiae s.s.* LL, and GG homozygous susceptible genotype (SS); LF, LS, and GS heterozygous genotype (RS); FF, and SS, homozygous resistant genotype (RR). The frequencies of resistance mutations were calculated using the formula: F=[(2RR+ RS)]/[2(RR+RS+SS)].

Discussion

This study investigated the *An. gambiae* complex members and resistance levels and mechanisms to pyrethroids (i.e., alpha-cypermethrin permethrin and deltamethrin) with and without PBO, clothianidin and chlorfenapyr in three coastal health districts of Aboisso, Jacqueville and San Pedro in southern Côte d'Ivoire. To our knowledge, this is one of the rare studies investigating both, the resistance status and complex members of malaria *Anopheles* mosquitoes in Costal Côte d'Ivoire. The current study showed that *An. gambiae s.l.* was composed of three sibling species, namely *An. coluzzii*, *An. gambiae* s.s and hybrids. Among the three sibling species *An. coluzzii* was the predominant (88.24%) species. The samples from Jacqueville and San Pedro were composed only of *An. coluzzii*. The three sibling species were represented in the Aboisso *An. gambiae s.l.* population. Among the sibling species *An. coluzzii*

Lines 78, 351-352, 347, 349, 351, 380...: Please consider revising these sentences and other as referring to the definition of species "Hybrids" individual cannot be considered as species.

Having this in mind revise also lines 238 and 364 as if not a species it cannot be part of the An. gambiae compex

Again, please be consistant with wording, consider "Vgsc" of "Kdr"

(66.95%) was most abundant followed by *An. gambiae* s.s and hybrids. Moreover, *An. gambiae s.l.* showed high phenotypic and genotypic resistance to pyrethroids, with increased mortality while pre-exposed to PBO. Overall, *An. gambiae s.l.* susceptibility was fully restored by clothianidin and chlorfenapyr throughout, except for the Aboisso population with clothianidin. *Vgsc* 995F allele frequency was very high (0.72-0.80%) in all *An. gambiae s.l.* populations. The *Vgsc* 995S was very low (0-0.06%) in all *An. gambiae s.l.* populations. For Ace-1 280S, the high frequency (0.57%) was recorded in San Pedro and the low (0) from the Aboisso hybrid population.

Please consider the tense, "...was..." instead of "...has been.."

line 362: Please consider removing "...excluding the presence of An. gambiae s.s. or hybrid..."

Species identification of *An. gambiae s.l.* showed that *An. coluzzii* constituted the major specie among *An. gambiae* complex. In San Pedro and Jacqueville, only the species *An. coluzzii* has been identified, excluding the presence of *An. gambiae s.s.* or hybrids. This selective distribution of vectors is of considerable importance for understanding malaria transmission in these regions. *Anopheles coluzzii*'s host preferences and specific breeding habitats, particularly its attraction to urban areas, may explain its dominance in these forested zones [36].

Lines 364 - 365: Contrasting inference here, I do not think An. coluzzii is linked to urban but the type of breeding sites as they breed preferentially in permanent water bodies such us puddles and farming sites.

In addition, I could not see this from the reference [36] you cite here

Furthermore, the geographical distribution of various *An. gambiae s.l.* species may result from local ecological variations and adaptations to specific microclimates at each site. In Aboisso, the dominance of *An. coluzzii* over *An. gambiae s.s.* and hybrids carries profound implications for malaria transmission and vector control [37]. *Anopheles coluzzii* prevalence underscores its role as the primary vector, given its affinity for human hosts and urban breeding sites, which enhance the efficiency of malaria transmission [36]. The lower prevalence of *An. gambiae s.s.* may be attributed to ecological factors or competition with *An. coluzzii* [12]. Hybrid mosquitoes, comprising 11.8% of the complex, introduce variables such as insecticide resistance and altered behavior [38,39]. Customizing vector control strategies, including the use of insecticide-treated bed nets and indoor spraying, to the dominant species, particularly *An. coluzzii*, is crucial [40]. The dominance of *An. coluzzii* species among *An. gambiae* complex is

commonly reported in southern Côte d’Ivoire [7,11,12,14,17,21]. Human activities such as urban Agriculture (vegetable gardens, irrigated rice fields) could have contributed to the development of the breeding sites of *An. gambiae s.l.* [24,41]. The presence of sibling species, namely *An. coluzzii*, *An. gambiae s.s.* and hybrids, which is consistent with previous studies in the same area [12,17]. Moreover, the presence of both *An. coluzzii* and *An. gambiae s.s.*, and the hybrids in the same area found here may imply that the mating appears to be occurring between the two species.

In the bioassays conducted in this study with pyrethroid, the highest mortalities were obtained with deltamethrin. Indeed, deltamethrin and alphacypermethrin were type II pyrethroids contrary to permethrin which is type I. Type II pyrethroids are distinguished from type I pyrethroids by the presence of a cyano group at the carbon of the esterified alcohol [42]. The effect of the cyano group on insecticidal activity increases mortality [43]. In such settings, deltamethrin or alphacypermethrin used to treat nets would probably be a good choice. Several studies have shown the performance of type II pyrethroids in comparison to Type I [44]. However, the mortality varied between both insecticides. Several reasons would explain deferred mortalities induced by the same insecticide in different years. Pyrethroid resistance was detected more than 30 years ago in malaria vectors (*An. gambiae s.l.*.) in Côte d'Ivoire for the first time in vectors from the Bouaké population [45]. In this study, *An. gambiae s.l.* from Aboisso, Jacqueville and San Pedro showed very strong phenotypic resistance to pyrethroids resulting in a very low mortality at standard diagnosis doses. The result is consistent with the general trends of *An. gambiae s.l.* resistance reported by previous studies in Côte d’Ivoire [15,17] and reinforces the view that Côte d'Ivoire is a hotspot of resistance in West Africa region [13,15,17]. The phenotypic resistance to pyrethroids may imply the presence of genetic resistance involving insecticide target site modification due to the use of the same insecticides

Line 385: Please consider using “are” instead

lines 399 - 401: Do you mean, that the use of the same insecticides in bot agriculture and public health lead to target site mutations?

Surprising, what causes the other resistance mechanisms then.

I doubt, please make your idea clearer here.

Line 403: Pesticide is a genral term of chemical that kills pests and insecticide is a types of pesticides used against insects. Thus, please, revise the following "...including pesticides and insecticides..."

in both agriculture and public health [22–24]. The coastal zones in southern Côte d'Ivoire are devoted to large agro-industrial units (cocoa, rubber and oil palm plantations [20,22,46]) that are treated with chemicals, including pesticides and insecticides to protect the crops [14, 33-35]. Indeed, the massive and incorrect use of pesticides by untrained farmers can contribute to the development of multiple resistance of *An. gambiae s.l.* against insecticides used in public health [48].

In this study, very high allelic frequencies of the L995F *kdr* gene (>38%) were found in *An. gambiae s.l.* from the coastal health districts of Aboisso, Jacquevile and San Pedro. These high allele frequencies could reveal the widespread of L995F and kdr-West mutation within the local *An. gambiae s.l.*., as found in several regions of Côte d'Ivoire [12, 14, 15]. The low allelic frequencies of the L995S *kdr* gene (<6%) were detected in local *An. gambiae s.l.* The 995S allele was only found in *An. gambiae s.s.* from Aboisso and *An. coluzzii* from Jacqueville. Nonetheless, the presence of this allele can deliver further evidence for a pan-African propagation of the kdr resistance. This spread of the *kdr* 995F and 995S gene in *An. gambiae*

line 416: please revise the sentence, it is not clear enough to me.

s.l. populations in littoral could compromise the effectiveness of the vector control tools that are currently in use. If the *kdr* 995F and 995S gene (against pyrethroids) spread, the efficacy of the malaria vector control tools (LLINs and IRS) was lost [49]. The high frequencies of G119S

Lines 408- 410 and 417 - 419: there is a kind of redundancy here,, "high frequency" obviously means "high prevalence"

mutation in Aboisso and Jacqueville may reveal a large prevalence of this mutation in coastal areas of Côte d'Ivoire. *Ace-1* G119S allele confers resistance to organophosphates and carbamates that are commonly used in Côte d'Ivoire for crop protection [14, 35].

The present study shows that an increase in insecticide concentrations and PBO-pre-exposure significantly increased mortality in all three coastal populations of *An. gambiae s.l.* against all pyrethroids, but did not fully restore the susceptibility. Indeed, the current study showed that high intensity resistance is present in all three *An. gambiae s.l.* populations, with overall

Line 427 - 428: the sentence is not clear enough, how could a “reduced insectidal activity” be associated with “lower personal protection”

Lines 428 and 430: Please consider revising the citation [29] as i suggested earlier

mortality being under 98% at 1X, 5X and 10X for all pyrethroid insecticides as reported by Kouassi [17] who carried out similar tests in Southern and central districts of Cote d’Ivoire. Such reduced insecticidal activity could result in lower personal protection [50], thus threatening the effectiveness of LLINs [29]. According to WHO, the vector control operational failure is likely to happen if resistance is confirmed at the 5X and especially at the 10X concentrations [29]. The various anthropogenic or natural xenobiotics present in local mosquito breeding sites and the biotic interaction could lead to the development of very high levels of resistance observed in the study [51]. The high intensity resistance recorded using only pyrethroid could be explained by high kdr mutation frequency, mixed function oxidases (MFO) activities, and an increase in esterase activity. However, moderate resistance intensity was recorded with a 10X concentration of alphacypermethrin in the Aboisso populations, and additional investigations are required for better understanding. In this study, pre-exposure to PBO showed significantly increased pyrethroid-induced mortality in all *An. gambiae s.l.* populations. However, full susceptibility was not recovered using PBO, except with the strain of Jacqueville with deltamethrin. Indeed, mortalities in PBO-pre-exposed mosquitoes tested against pyrethroids were still lower than the WHO susceptibility cut-off, as observed in other areas in Côte d'Ivoire [17,52]. Significant increase in mortality after exposure to PBO may indicate the presence of metabolic resistance (MFO, P450s, and esterases) [52], and this can compromise the effectiveness of non-PBO LLINs (e.g., Panda.Net, MagNet, PermaNet 2.0). In addition, non-restoration of full susceptibility with PBO might reduce the efficacy of PBO-treated LLINs (e.g., PermaNet 3.0) [53].

Line 437: Please consider “...significant...” instead

Plsease consider revisiting lines 421 - 423 and lines 436 - 438 as they seem to bring the same information

In contrast to PBO, chlorfenapyr and clothianidin restored fully the susceptibility in all populations of *An. gambiae s.l.* from Aboisso, Jacqueville and San Pedro, except for clothianidin in Aboisso population in the current study. The pyrrole chlorfenapyr and neonicotinoid clothianidin have new modes of action and are good candidates for malaria vector

Line 448: please revise the term “...pyrrole chlorfenapyr...neonicotinoid clothianidin”

control in areas comprised of mutation and/or metabolic resistance to pyrethroids [54]. Indeed, after exposure to chlorfenapyr, full susceptibility was recovered in one of three districts at 100µg ai/bottle. However, when the samples were tested with higher dosages (200µg ai/bottle), the mortality increased, and susceptibility was restored in all districts. Chlorfenapyr requires activation, via cytochrome P450 monooxygenases [55]. Chlorfenapyr-treated LLINs (e.g., Interceptor G2 and PermaNet Dual) could be effective against *An. gambiae s.l.* in coastal Côte d'Ivoire [3,4,54]. Moreover, *An. gambiae s.l.* susceptibility was restored with clothianidin (100% mortality) in Jacqueville and San Pedro. As the National Malaria Strategic Plan of the National Malaria Control Programme of Côte d'Ivoire prioritizes indoor residual spraying (IRS), using clothianidin-based IRS (e.g., Fludora® Fusion) could be effective against the coastal *An. gambiae s.l.* populations in coastal Côte d'Ivoire.

Conclusion

The present study showed that *An. gambiae s.l.* was mainly composed of *An. coluzzii*, followed by *An. gambiae* s.s and hybrids in costal Côte d'Ivoire. Local *An. gambiae s.l.* populations were highly resistant to pyrethroids and susceptible to clothianidin and chlorfenapyr. PBO pre-exposure increased the mortality, but susceptibility was not fully recovered. The coastal *An. gambiae s.l.* populations had high frequencies of target site mutation genes (kdr-West and kdr-East) and metabolic genes (Ace-1). Clothianidin and chlorfenapyr did restore full susceptibility in *An. gambiae s.l.* populations., suggesting that vector control tools treated with these new insecticides in costal Côte d'Ivoire.

Lines 465 - 466: please consider "Pre-exposure to PBO…" instead

Sentence in lines 468 - 470 need revision as missing the main verb and complement.

Author Contributions

Jackson Koffi Ives Kouamé: Conceptualization, Methodology, Investigation, Visualization, Data curation, Formal analysis, Writing – review & editing**. Ako Constant Victorien Edi :** Conceptualization, Methodology, Supervision**. Benjamin Guibehi Koudou**

Conceptualization, Methodology, Supervision, Visualization, Writing – review & editing**. Ruth Marie Adjoua Kouamé:** Formal analysis, Writing – review & editing**. Yves Alain Kadio Kacou:** Investigation. **Firmain N'dri Yokoly** Writing – review & editing. Visualization**: Julien Bi Zahouli Zahouli** Methodology, Writing – review & editing**. Constant Guy N'Guessan Gbalegba :** Writing – review & editing. **David Malone :** Writing – review & editing.

Acknowledgements

We gratefully acknowledge the contribution of technician Assamoi Jean Baptiste of the Centre Suisse de Recherches Scientifiques en Côte d'Ivoire for help and assistance during laboratory analysis. We also thank the local health district staff and a special thanks to the Programme National de Lutte contre le Paludisme Côte d'Ivoire for facilitating and participating in field data collection.

Conflict of interest statement: The author declares that there are no conflicts of interest.

ORCID

Jackson Koffi Ives Kouamé : https://orcid.org/0009-0005-1469-0481

Ako Constant Victorien Edi: https://orcid.org/0000-0003-1564-4354

Julien Bi Zahouli Zahouli : https://orcid.org/0000-0002-2351-4309

References

1. WHO. World malaria report 2023. Geneva: World Health Organization; 2023. Available: https://reliefweb.int/attachments/f03b4f2c-af9a-4161-9ee2-f2cec1eb3dc8/World%20malaria%20report%202023.pdf

2. WHO. World malaria report 2022. Geneva: World Health Organization; 2022. Available: https://www.who.int/publications/i/item/9789240064898

3. Oxborough RM, Seyoum A, Yihdego Y, Chabi J, Wat'senga F, Agossa FR, et al. Determination of the discriminating concentration of chlorfenapyr (pyrrole) and Anopheles gambiae sensu lato susceptibility testing in preparation for distribution of Interceptor® G2 insecticide-treated nets. Malar J. 2021;20: 316. doi:10.1186/s12936-021-03847-3

4. Oxborough RM, N'Guessan R, Jones R, Kitau J, Ngufor C, Malone D, et al. The activity of the pyrrole insecticide chlorfenapyr in mosquito bioassay: towards a more rational testing and screening of non-neurotoxic insecticides for malaria vector control. Malar J. 2015;14: 124. doi:10.1186/s12936-015-0639-x

5. Institut National de la Statistique (INS). Enquête de prévalence parasitaire du paludisme et de l'anémie 2016. 2016; 61.

6. PRESIDENT'S MALARIA INITIATIVE. U.S. President's Malaria Initiative Côte d'Ivoire Malaria Operational Plan FY 2023. 2023; 56.

7. Kouassi BL, Edi C, Ouattara AF, Ekra AK, Bellai LG, Gouaméné J, et al. Entomological monitoring data driving decision-making for appropriate and sustainable malaria vector control in Côte d'Ivoire. Malar J. 2023;22: 14. doi:10.1186/s12936-023-04439-z

8. Ouattara AF, Dagnogo M, Constant EA, Koné M, Raso G, Tanner M, et al. Transmission of malaria in relation to distribution and coverage of long-lasting insecticidal nets in central Côte d'Ivoire. Malar J. 2014;13: 109. doi:10.1186/1475-2875-13-109

9. N'Guessan GKD, Coulibaly FH, Barreaux AMG, Yapo RJ, Adou KA, Tia E, et al. Qualitative study on the use and maintenance of long-lasting insecticidal nets (LLINs) in

Bouaké (Côte d'Ivoire), 17 months after the last mass distribution campaign. Malar J. 2022;21: 228. doi:10.1186/s12936-022-04243-1

10. The PMI VectorLink Côte d'Ivoire 2021 Annual Entomological Report, January–December 2021. : 65.

11. Assouho KF, Adja AM, Guindo-Coulibaly N, Tia E, Kouadio AMN, Zoh DD, et al. Vectorial Transmission of Malaria in Major Districts of Côte d'Ivoire. Norris D, editor. Journal of Medical Entomology. 2020;57: 908–914. doi:10.1093/jme/tjz207

12. Yokoly FN, Zahouli JBZ, Small G, Ouattara AF, Opoku M, de Souza DK, et al. Assessing Anopheles vector species diversity and transmission of malaria in four health districts along the borders of Côte d'Ivoire. Malar J. 2021;20: 409. doi:10.1186/s12936-021-03938-1

13. Kouamé RMA, Lynd A, Kouamé JKI, Vavassori L, Abo K, Donnelly MJ, et al. Widespread occurrence of copy number variants and fixation of pyrethroid target site resistance in Anopheles gambiae (*s.l.*.) from southern Côte d'Ivoire. Current Research in Parasitology & Vector-Borne Diseases. 2023;3: 100117. doi:10.1016/j.crpvbd.2023.100117

14. Fodjo BK, Koudou BG, Tia E, Saric J, N'dri PB, Zoh MG, et al. Insecticides Resistance Status of *An. gambiae* in Areas of Varying Agrochemical Use in Côte D'Ivoire. BioMed Research International. 2018;2018: 1–9. doi:10.1155/2018/2874160

15. Camara S, Koffi AA, Ahoua Alou LP, Koffi K, Kabran J-PK, Koné A, et al. Mapping insecticide resistance in Anopheles gambiae (*s.l.*.) from Côte d'Ivoire. Parasites Vectors. 2018;11: 19. doi:10.1186/s13071-017-2546-1

16. Edi CA, Koudou BG, Bellai L, Adja AM, Chouaibou M, Bonfoh B, et al. Long-term trends in Anopheles gambiae insecticide resistance in Côte d'Ivoire. 2014; 10.

17. Kouassi BL, Edi C, Tia E, Konan LY, Akré MA, Koffi AA, et al. Susceptibility of Anopheles gambiae from Côte d'Ivoire to insecticides used on insecticide-treated nets: evaluating the additional entomological impact of piperonyl butoxide and chlorfenapyr. Malar J. 2020;19: 454. doi:10.1186/s12936-020-03523-y

18. PRESIDENT'S MALARIA INITIATIVE. U.S. President's Malaria Initiative Côte d'Ivoire Malaria Operational Plan FY 2018 and FY 2019. 2018; 57.

19. MSCMU. Rapport annuel sur la situation sanitaire (RASS). Côte d'Ivoire; 2017. 2017; 414.

20. Anoh P. Anoh Kouassi Paul et Pottier Patrick, dirs., 2008 - Géographie du littoral de Côte-d'Ivoire : éléments de réflexion pour une politique de gestion inté. com. 2010;63: 485–489. doi:10.4000/com.6097

21. Sadia-Kacou CMA, Alou LPA, Edi AVC, Yobo CM, Adja MA, Ouattara AF, et al. Presence of susceptible wild strains of Anopheles gambiae in a large industrial palm farm located in Aboisso, South-Eastern of Côte d'Ivoire. Malar J. 2017;16: 157. doi:10.1186/s12936-017-1804-1

22. Gohourou F, Ahua ÉA, Gnanbe DR, Desse M. Fonctionnement, dynamique et structuration de l'espace littoral en Côte d'Ivoire. : 16.

23. Malan DF, Neuba DFR, Kouakou KL. Medicinal plants and traditional healing practices in ehotile people, around the aby lagoon (eastern littoral of Côte d'Ivoire). J Ethnobiology Ethnomedicine. 2015;11: 21. doi:10.1186/s13002-015-0004-8

24. Chouaïbou MS, Fodjo BK, Fokou G, Allassane OF, Koudou BG, David J-P, et al. Influence of the agrochemicals used for rice and vegetable cultivation on insecticide resistance in malaria vectors in southern Côte d'Ivoire. Malar J. 2016;15: 426. doi:10.1186/s12936-016-1481-5

25. Gillies MT, Meillon B de. The Anophelinae of Africa south of the Sahara. Suppl: Afrotropical region. Johannesburg; 1987.

26. WHO. World malaria report : 2013. Geneva: World Health Organization; 2013. Available: https://apps.who.int/iris/handle/10665/97008

27. WHO. Malaria control improves for vulnerable in Africa, but global progress off-track = La lutte contre le paludisme s'améliore pour les personnes vulnérables en Afrique, mais les progrès stagnent à l'échelle mondiale. Is part of a journal: Weekly Epidemiological Record = Relevé épidémiologique hebdomadaire. 2016;91: 622–624.

28. Dagg K, Irish S, Wiegand RE, Shililu J, Yewhalaw D, Messenger LA. Evaluation of toxicity of clothianidin (neonicotinoid) and chlorfenapyr (pyrrole) insecticides and cross-resistance to other public health insecticides in Anopheles arabiensis from Ethiopia. Malar J. 2019;18: 49. doi:10.1186/s12936-019-2685-2

Please add the link as you did for the other refs

29. WHO. Weekly Epidemiological Record, 2016, vol. 91, 51/52 [full issue]. Weekly Epidemiological Record = Relevé épidémiologique hebdomadaire. 2016;91: 601–624.

30. Collins FH, Besansky NJ, Mendez MA, Rasmussen MO, Finnerty V, Mehaffey PC. A Ribosomal RNA Gene Probe Differentiates Member Species of the Anopheles gambiae Complex. The American Journal of Tropical Medicine and Hygiene. 1987;37: 37–41. doi:10.4269/ajtmh.1987.37.37

31. Santolamazza F, Calzetta M, Etang J, Barrese E, Dia I, Caccone A, et al. Distribution of knock-down resistance mutations in Anopheles gambiae molecular forms in west and west-central Africa. Malar J. 2008;7: 74. doi:10.1186/1475-2875-7-74

32. Lucas ER, Rockett KA, Lynd A, Essandoh J, Grisales N, Kemei B, et al. A high throughput multi-locus insecticide resistance marker panel for tracking resistance emergence and spread in Anopheles gambiae. Sci Rep. 2019;9: 13335. doi:10.1038/s41598-019-49892-6

33. Bass C, Nikou D, Donnelly MJ, Williamson MS, Ranson H, Ball A, et al. Detection of knockdown resistance (kdr) mutations in Anopheles gambiae: a comparison of two new high-throughput assays with existing methods. Malar J. 2007;6: 111. doi:10.1186/1475-2875-6-111

34. Abbott WS. A Method of Computing the Effectiveness of an Insecticide. Journal of Economic Entomology. 1925;18: 265–267. doi:10.1093/jee/18.2.265a

35. Hardy GH. Mendelian Proportions in a Mixed Population. Science. 1908;28: 49–50. doi:10.1126/science.28.706.49

36. Gimonneau G, Pombi M, Choisy M, Morand S, Dabiré RK, Simard F. Larval habitat segregation between the molecular forms of the mosquito Anopheles gambiae in a rice field area of Burkina Faso, West Africa. Medical and Veterinary Entomology. 2012;26: 9–17. doi:10.1111/j.1365-2915.2011.00957.x

37. Smith DL, Battle KE, Hay SI, Barker CM, Scott TW, McKenzie FE. Ross, Macdonald, and a Theory for the Dynamics and Control of Mosquito-Transmitted Pathogens. Chitnis CE, editor. PLoS Pathog. 2012;8: e1002588. doi:10.1371/journal.ppat.1002588

38. Norris LC, Main BJ, Lee Y, Collier TC, Fofana A, Cornel AJ, et al. Adaptive introgression in an African malaria mosquito coincident with the increased usage of insecticide-treated bed nets. Proc Natl Acad Sci USA. 2015;112: 815–820. doi:10.1073/pnas.1418892112

39. Caputo B, Tondossoma N, Virgillito C, Pichler V, Serini P, Calzetta M, et al. Is Côte D'Ivoire a new high hybridization zone for the two major malaria vectors, Anopheles coluzzii and An. gambiae (Diptera, Culicidae)? Infection, Genetics and Evolution. 2022;98: 105215. doi:10.1016/j.meegid.2022.105215

40. Moyes CL, Vontas J, Martins AJ, Ng LC, Koou SY, Dusfour I, et al. Contemporary status of insecticide resistance in the major Aedes vectors of arboviruses infecting humans. Sinnis P, editor. PLoS Negl Trop Dis. 2017;11: e0005625. doi:10.1371/journal.pntd.0005625

41. Chabi J, Eziefule MC, Pwalia R, Joannides J, Obuobi D, Amlalo G, et al. Impact of Urban Agriculture on the Species Distribution and Insecticide Resistance Profile of <i>Anopheles gambiae *s.s.*</i> and <i>Anopheles coluzzii</i> in Accra Metropolis, Ghana. AE. 2018;06: 198–211. doi:10.4236/ae.2018.63016

42. Godin SJ, Crow JA, Scollon EJ, Hughes MF, DeVito MJ, Ross MK. Identification of Rat and Human Cytochrome P450 Isoforms and a Rat Serum Esterase That Metabolize the Pyrethroid Insecticides Deltamethrin and Esfenvalerate. Drug Metab Dispos. 2007;35: 1664–1671. doi:10.1124/dmd.107.015388

43. Kobayashi T, Nishimura K, Fujita T. Effects of the α-cyano group in the benzyl alcohol moiety on insecticidal and neurophysiological activities of pyrethroid esters. Pesticide Biochemistry and Physiology. 1989;35: 231–243. doi:10.1016/0048-3575(89)90084-9

44. Dadzie SK, Chabi J, Asafu-Adjaye A, Owusu-Akrofi O, Baffoe-Wilmot A, Malm K, et al. Evaluation of piperonyl butoxide in enhancing the efficacy of pyrethroid insecticides against resistant Anopheles gambiae *s.l.*. in Ghana. Malar J. 2017;16: 342. doi:10.1186/s12936-017-1960-3

45. Elissa N, Mouchet J, Riviere F, Meunier JY, Yao K. Resistance of Anopheles gambiae *s.s.* to pyrethroids in Côte d'Ivoire. Ann Soc Belg Med Trop. 1993;73: 291–294.

46. Pottier P. GÉOGRAPHIE DU LITTORAL DE CÔTE D IVOIRE Éléments de réflexion pour une politique de gestion intégrée. 2008 [cited 16 May 2022]. Available: https://docplayer.fr/56603270-Geographie-du-littoral-de-cote-d-ivoire-elements-de-reflexion-pour-une-politique-de-gestion-integree.html

47. MINADER. PROGRAMME DE TRANSFORMATION DE L'AGRICULTURE EN AFRIQUE DE L'OUEST (PTAAO) PLAN DE GESTION DES PESTES (PGP). Abidjan; 2018 Apr p. 93. Available: http://www.waapp-ppaao.org/sites/default/files/ptaao-cote_divoire-document_de_suavegarde-plan_de_gestion_des_pestes.pdf

48. WHO, FAO. International code of conduct on pesticide management: guidance on management of household pesticides. Geneva: World Health Organization; 2020. Available: https://apps.who.int/iris/handle/10665/337126

49. Mouhamadou CS, N'Dri PB, Fodjo BK, Sadia CG, Affoue F-PK, Koudou BG. Rapid spread of double East- and West-African kdr mutations in wild Anopheles coluzzi from Côte d'Ivoire. Wellcome Open Res. 2019;4: 31. doi:10.12688/wellcomeopenres.15105.1

50. Dabiré RK, Diabaté A, Baldet T, Paré-Toé L, Guiguemdé RT, Ouédraogo J-B, et al. Personal protection of long lasting insecticide-treated nets in areas of Anopheles gambiae *s.s.* resistance to pyrethroids. Malar J. 2006;5: 12. doi:10.1186/1475-2875-5-12

51. Poupardin R, Riaz MA, Vontas J, David JP, Reynaud S. Transcription profiling of eleven cytochrome P450s potentially involved in xenobiotic metabolism in the mosquito *Aedes aegypti*. Insect Molecular Biology. 2010;19: 185–193. doi:10.1111/j.1365-2583.2009.00967.x

52. Bingham G, Strode C, Tran L, Khoa PT, Jamet HP. Can piperonyl butoxide enhance the efficacy of pyrethroids against pyrethroid-resistant Aedes aegypti?: Can piperonyl butoxide enhance the efficacy of pyrethroids? Tropical Medicine & International Health. 2011;16: 492–500. doi:10.1111/j.1365-3156.2010.02717.x

53. Gleave K, Lissenden N, Chaplin M, Choi L, Ranson H. Piperonyl butoxide (PBO) combined with pyrethroids in insecticide-treated nets to prevent malaria in Africa. Cochrane Infectious Diseases Group, editor. Cochrane Database of Systematic Reviews. 2021;2021. doi:10.1002/14651858.CD012776.pub3

54. N'Guessan R, Odjo A, Ngufor C, Malone D, Rowland M. A Chlorfenapyr Mixture Net Interceptor® G2 Shows High Efficacy and Wash Durability against Resistant Mosquitoes in West Africa. Vontas J, editor. PLoS ONE. 2016;11: e0165925. doi:10.1371/journal.pone.0165925

55. Paul A, Harrington LC, Scott JG. Evaluation of Novel Insecticides for Control of Dengue Vector <I>Aedes aegypti</I> (Diptera: Culicidae). me. 2006;43: 55–60. doi:10.1603/0022-2585(2006)043[0055:EONIFC]2.0.CO;2

Figure 1

Click here to access/download;Figure;Figure1.tif

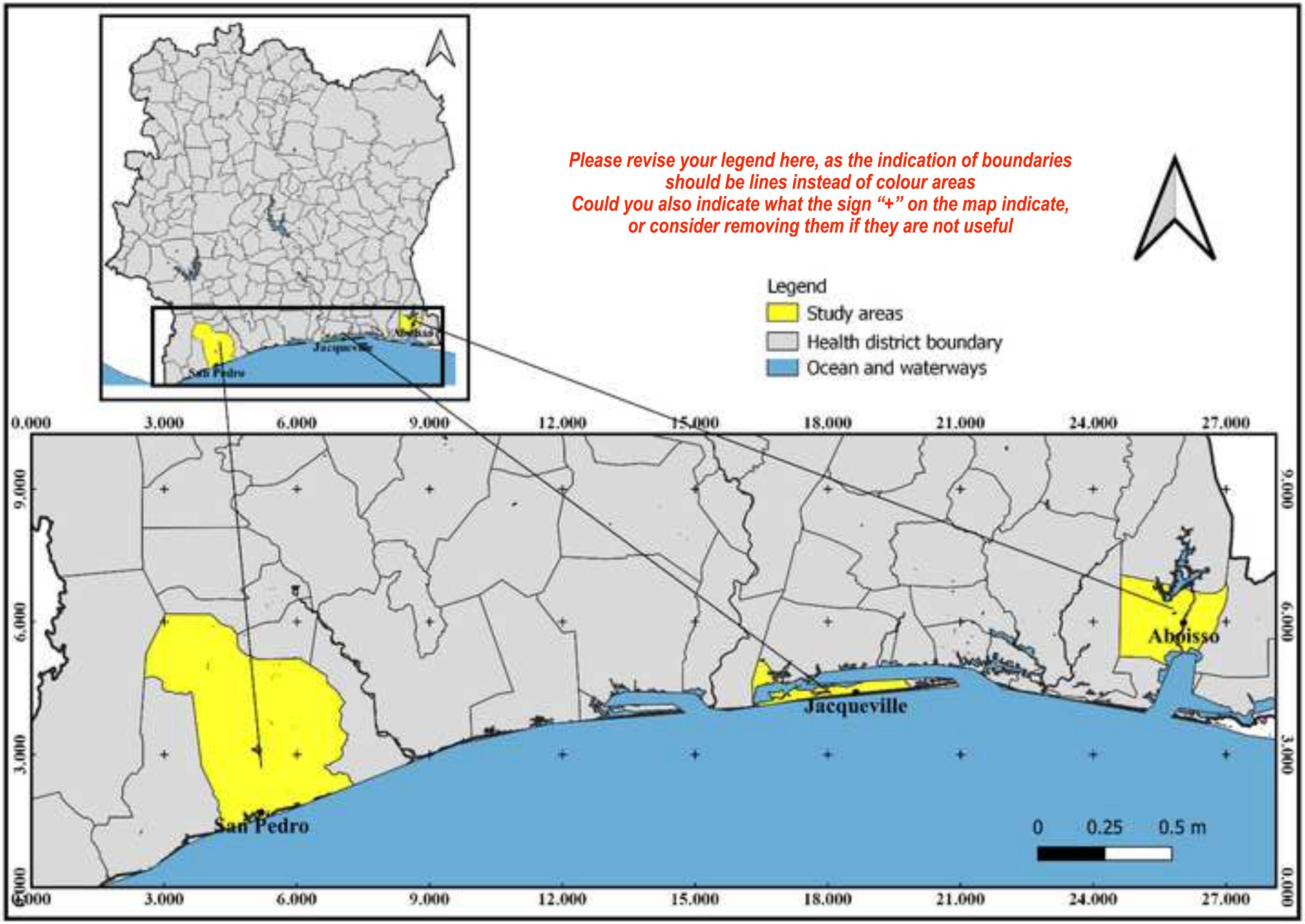

Figure 2

Click here to access/download;Figure;Figure2.tif

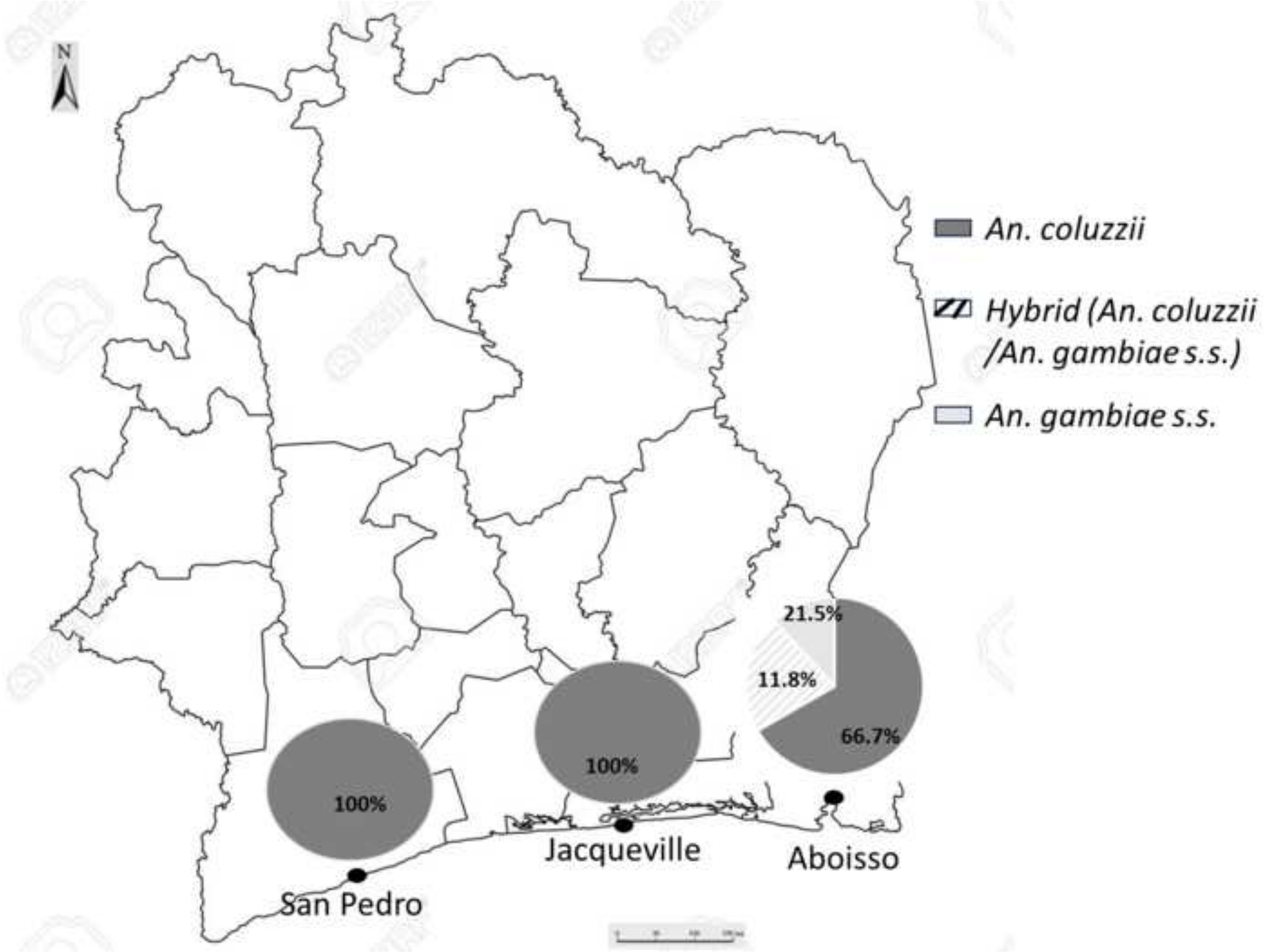

Figure 3

Click here to access/download;Figure;Figure3.tif

the quality of the figure 3 to 6 and their legends are very poor and blurry, they need improvement

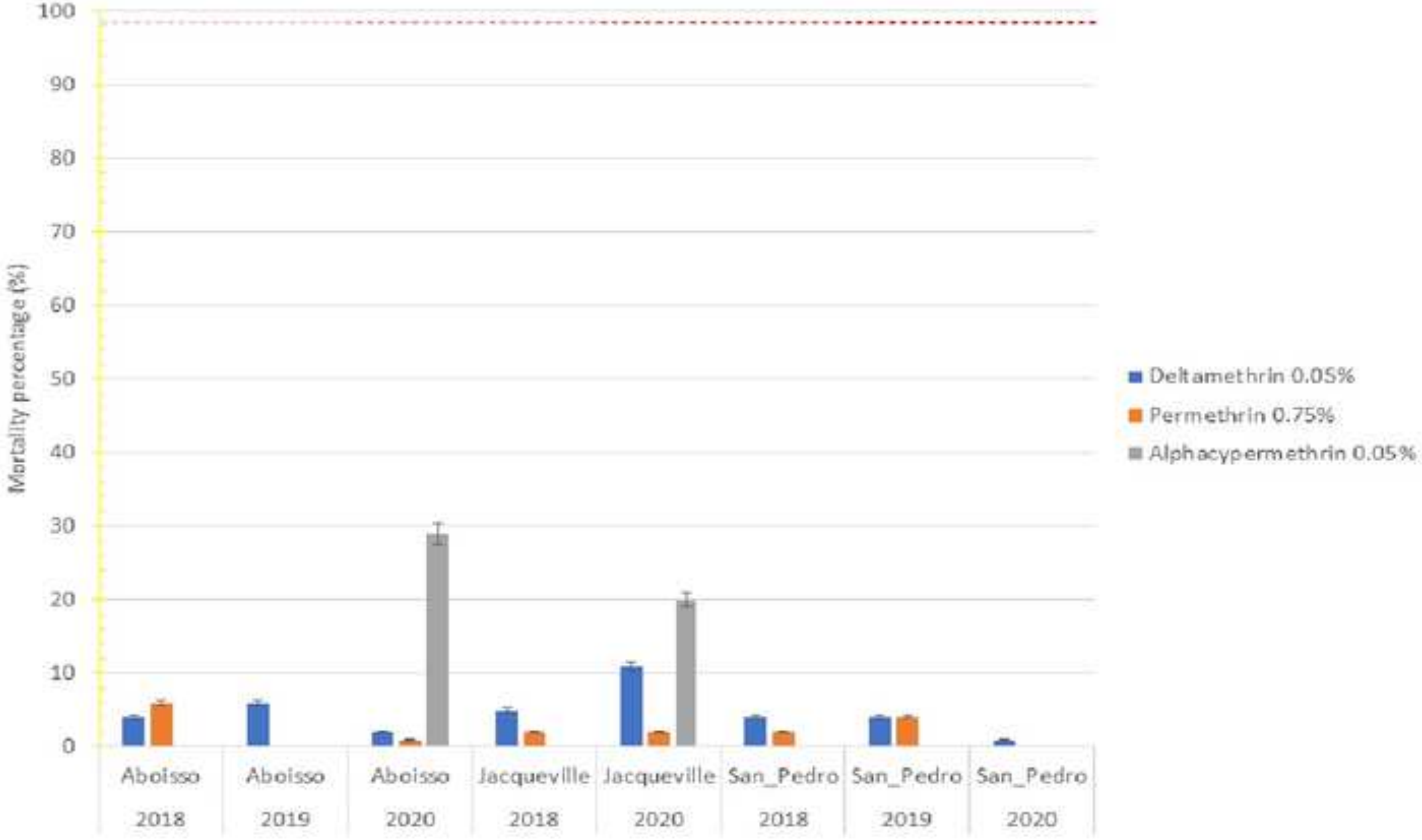

Figure 4

Click here to access/download;Figure;Figure4.tif

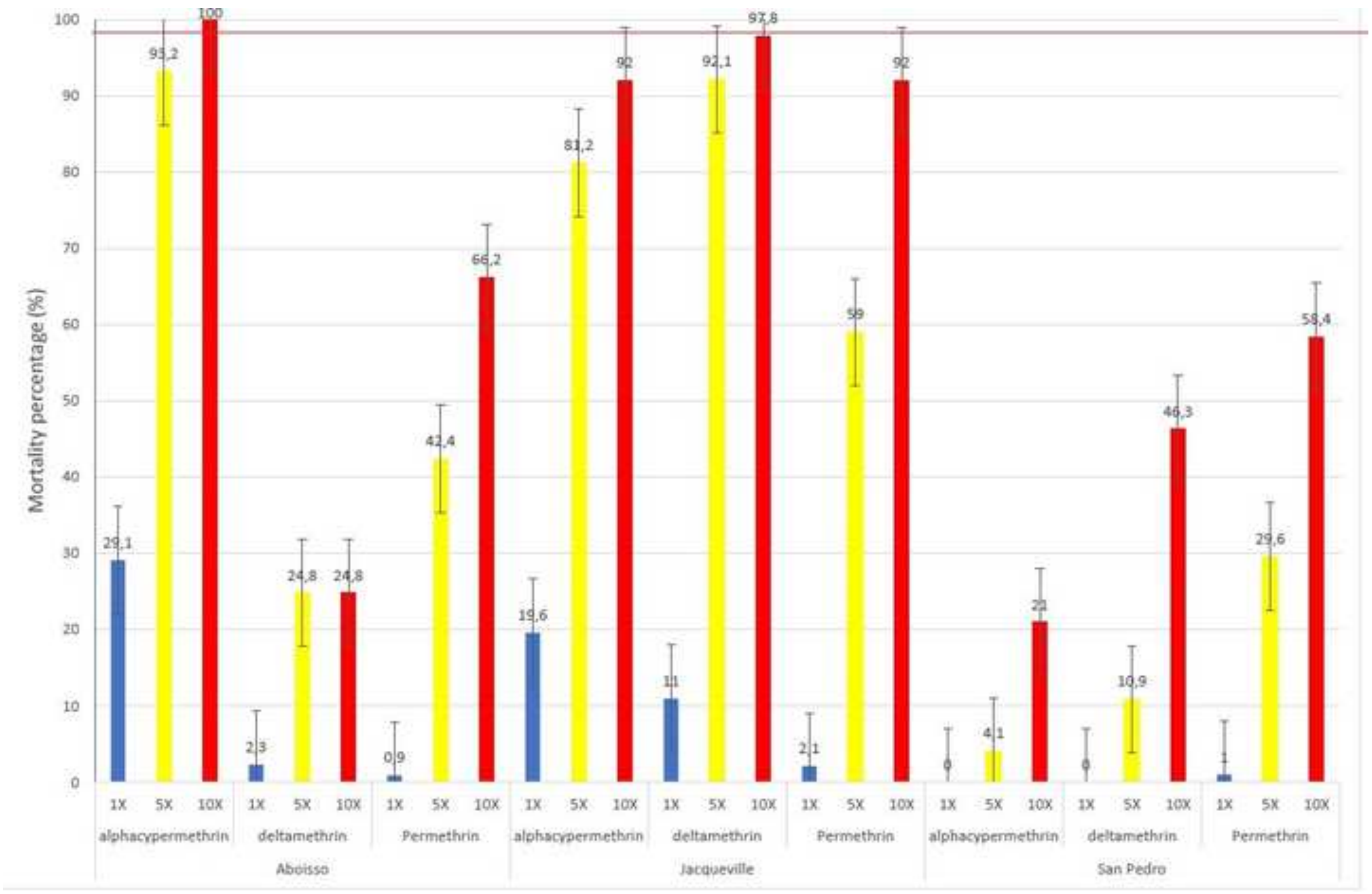

Figure 5

Click here to access/download;Figure;Figure5.tif

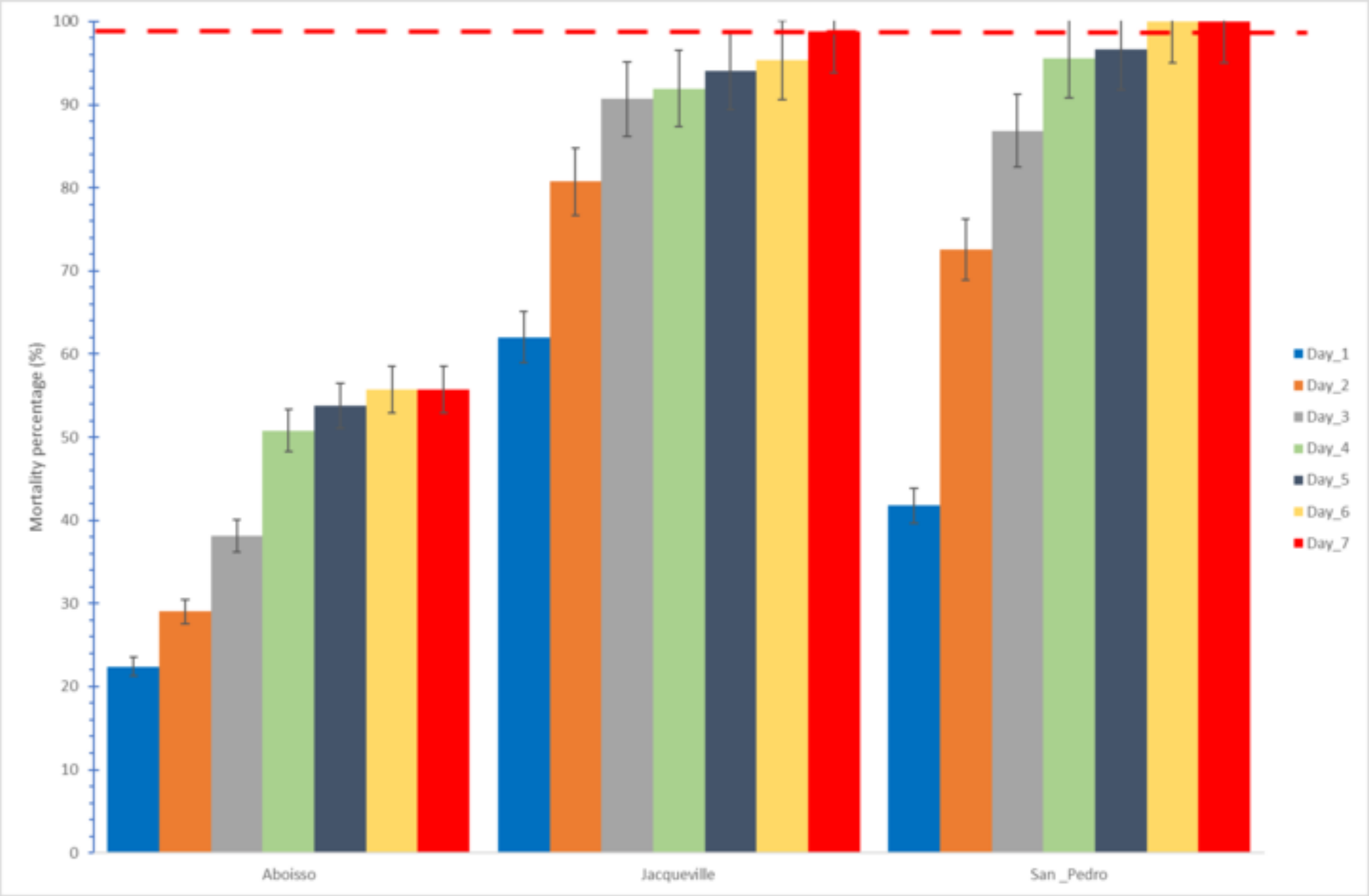

Figure 6

Click here to access/download;Figure;Figure6.tif

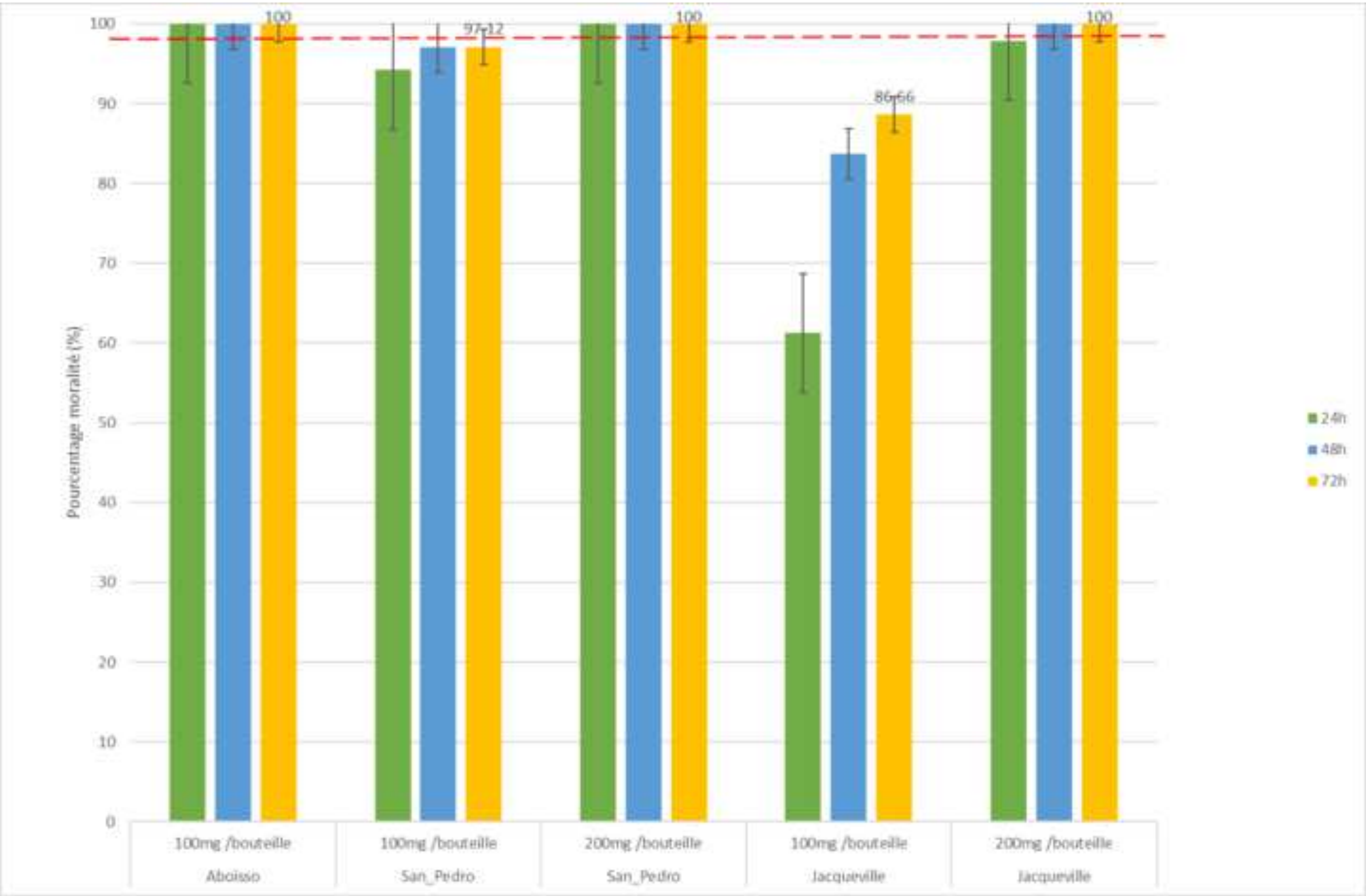

Table 1 Click here to access/download;Figure;Table1.docx

Table 1: Bioassays of *An. gambiae s.l.* to pyrethroids without and with piperonyl butoxide of the study population.

**Populations**	**Insecticide**	**N tested (Dead)**	**Mortality percentage (95% CI)**	**p-value**
Aboisso	Alphacypermethrin	82 (24)	29.1 (19.73-40.35)	<0.0001
	PBO+Alphacypermethrin	76 (72)	94.7 (87.07-98.55)	
	Deltamethrin	79 (2)	2.3 (0.31-8.85)	<0.0001
	PBO + Deltamethrin	84 (42)	50.0 (38.88-61.11)	
	Permethrin	105 (1)	0.9 (0.02-5.19)	<0.0001
	PBO + Permethrin	86 (28)	32.5 (22.84-43.52)	
Jacqueville	Alphacypermethrin	89 (18)	19.6 (12.45-30.07)	<0.0001
	PBO+Alphacypermethrin	82 (74)	90.1 (81.68-95.69)	
	Deltamethrin	90 (10)	0 (5.46-19.48)	<0.0001
	PBO + Deltamethrin	71 (70)	98.5 (92.40-99.96)	
	Permethrin	79 (2)	2.1 (0.31-8.85)	<0.0001
	PBO + Permethrin	68 (22)	31.7 (21.51-44.79)	
San Pedro	Alphacypermethrin	101 (0)	0 (0-3.59)	<0.0001
	PBO+Alphacypermethrin	106 (19)	17.9 (11.15-26.57)	
	Deltamethrin	94 (0)	0 (0-3.85)	<0.0001
	PBO + Deltamethrin	98 (28)	28.3 (19.90-38.58)	
	Permethrin	93 (1)	1.1 (0.03-05.85)	<0.0001
	PBO + Permethrin	103 (11)	10.6 (5.45-18.31)	

N=represents the total number of mosquitos tested, CI=represents the confidence intervals, and p-value were calculated using the prop.test.

Table 1: *An. gambiae s.l.* sibling species composition and *vgsc-995F*, *vgsc-995S* and *Ace-1 280S* frequency in three populations

Populations	***An. gambiae s.l.* molecular form**	***Kdr-West (L995F)***					***Kdr-East (L995S)***					***Ace-1 (G280S)***				
		**Tested**	**FF**	**LF**	**LL**	**%Fr (F)**	**Tested**	**SS**	**LS**	**LL**	**%Fr (S)**	**Tested**	**SS**	**GS**	**GG**	**%Fr (S)**
Aboisso	*An. coluzzii*	127	83	26	12	79.3	127	0	0	127	0	31	5	7	19	27.4
Aboisso	Hybrids	25	16	6	3	76.0	25	0	0	25	0	2	0	0	2	0
Aboisso	*An. gambiae s.s.*	44	23	16	4	72.1	44	0	2	42	2.3	7	3	0	4	42.9
Jacqueville	*An. coluzzii*	145	86	45	14	38.1	140	1	14	125	5.7	82	4	43	35	31.1
San Pedro	*An. coluzzii*	99	57	27	15	71.2	100	0	0	100	0	108	0	9	99	4.2

Hybrids represented *An. coluzzii/ An. gambiae s.s.* LL, and GG homozygous susceptible genotype (SS); LF, LS, and GS heterozygous genotype (RS); FF, and SS, homozygous resistant genotype (RR). The frequencies of resistance mutations were calculated using the formula: F = [(2RR+ RS)] / [2(RR+RS+SS)].
