# Supplementary material for: Assessing species composition and insecticide resistance of Anopheles gambiae complex members in three coastal health districts of Côte d’Ivoire
Source: PLoS One. 2024 Dec 10;19(12):e0297604. doi: 10.1371/journal.pone.0297604 (PMC11630590; doi:10.1371/journal.pone.0297604)
Supplement: Attachment — Submitted filename: Reviewer.pdf [file pone.0297604.s003.pdf]

Reviewer name: Jacques Dollon MBAMA NTABI

This manuscript describes the results of **assessing complex member composition and insecticide resistance status of *Anopheles gambiae s.l.* in three coastal health districts of Côte d'Ivoire**. The paper appears to be methodologically sound, and the analyses are well described in the manuscript. The strength of the paper is the fact that the authors are able to compare between three health districts,

Comments 1: Line 1 and 2. It's will be important to reformulate the title of the manuscript
I'm suggested**:** Assessing of species composition and insecticide resistance of Anopheles gambiae complex member in three coastal health districts of Côte d'Ivoire

The aim of the study was to investigate the complex members and the insecticide resistance in the *Anopheles gambiae s.l.* populations in coastal Côte d'Ivoire.

a. **Abstract:**

You can choice to write in the manuscript between "An. gambiae s.L." and "An. gambiae complex". It's a totology when you write *An. gambiae* s.L. complex

Comment 1: Line 33: Author should put the 4.7% of hybrid species for what species?
Comment 2: Author should put the abbreviation (*An. gambiae* s.L) throughout the abstract subsections as the species name "*Anopheles gambiae sensu lato"* was already mentioned at the beginning of the abstract in line 41.

Comment 3: Abstract and throughout the manuscript, common Latin terms such as "s.s" and "s.L" are not italicized.

Comment 4: The authors are not mentioned in the abstract and the methodology if this is a cross-sectional survey, a longitudinal survey…etc. Add in the abstract and the methodology (Study site)

b. **Introduction:**

Ccomment 1: This is a study of the An. gambiae complex, except that I have only seen two species of the complex mentioned in the manuscript. The authors needs to define the *An. gambiae* complex and to cite the nine member of *An. gambiae s. L*. These articles will help you to cite the different

species of the *An. gambiae* complex member, those which contribute to transmission and those which do not contribute. Loughlin SO (2020) https://doi.org/10.1080/20477724.2020.1722434. Abel *et al. (2024)* https://doi.org/10.1186/s13071-023-06102-7

Comment 2: The authors are not mentioned the Entomological Inoculation Rate (EIR) of *An. gambiae* s.L in coastal Côte d'Ivoire (in the studied sites).

c. **Methodology:**

Comment 1: Line 126 and 135. The larval method collected is not mentioned for what? What do you collected in the water wells, natural breeding sites, gardens field …etc larval only or not?

Comment 2: For rearing adult, author need to mention the technics used of rearing adult mosquito and the water type

Comment 3: Line 136**:** WHO tube bioassay assesses the degree of susceptibility for what?

Comment 4, Line 137 to 139: The author mentioned that the diagnostic dose tests of pyrethroids (alphacypermethrin, deltamethrin, and permethrin) were conducted in 2018, 2019, and 2020. The following tests were conducted exclusively in 2020 on the same population of An. gambiae complex (F_o, F_1, $F_{2...}$)?

Comment 5: The bottle analysis method is a monitoring tool for detecting and characterizing changes in insecticide susceptibility in vector populations?

Comment 6: Mosquito sample processing for DNA extraction was not mentioned clearly, does the author used whole mosquito for extraction or it was dissected to head and thorax and abdomen?

Identification of target site mutation (L202)

Comment 7: L202: For molecular Identification of target site mutation, author need to mention the primers used and PCR probes sequences and brief about the PCR methods.

Comment 8: L205-205: The author said that the krd (Kdr-East and Kdr-West) mutation was investigated using real-time PCR (TaqMan assay). To my knowledge, the probes (Kdr-East and West) attach to the FAM, I need to know how do you differentiate it? Did you do two separate the

mix-PCR (WT, sensimix, water and Kdr-E FAM; WT, sensimix, water and Kdr-W FAM) or in the same mix did you put the Kdr-E and Kdr-W?

Data analysis

Comment 9: Please explain how this statistical analysis plan answer the stated question,: role of individual members of *An. gambaie* compex to malaria transmission?

Comment 10: Were there any considerations for possible non-blood-fed females from exophagic, zoophagic and endophilic mosquitoes? Any chance that some mosquitoes feed?

d. **Results:**

Species composition of the *Anopheles gambiae* complex

Comment 1, L236: For molecular identification of *Anopheles gambiae complex member* species, author mentioned the SINE200 or SINE-PCR using **(Line 187 to 188).** To my knowledge, SINE-PCR makes it possible to identify three species of the An. gambiae complex, namely: *An. gambiae ss, An. arabesnsis and An. coluzzii*. This is what I don't see in this manuscripts, in these results, the author identify two species and the hybride, were these others species in the complex member?

Comment 2: L237: the author said that 672 *An. gambiae s.l.* was selected from the three studied sites for species identification, however only 671 samples were used in *An. gambaie s.l* species molecular identification (there is 1 sample undetectable), because when I do the calculations of 593+51+27=671, where is the remaining 1 mosquito?

Comment 3: Figure 2 is not adopted for these results, remove that. It is better to represent the distribution of *An. gambiae s.l.* species on a table

Comment 4: L244, Remove the "of members" and write in the Table XX: Distribution of *Anopheles gambiae s.l.* in the three studied sites

Insecticide susceptibility in *An. gambiae s.l*

Comment 5: How many Anopheles have been testing with each insecticide, years and the different studied sites? The number of each An. gambiae s.L. alive and death (e.g: 2% (20/672))?

Intensity of resistance section

Comment 6, L273-273: The author said that: these results confirmed the strong resistance of *An. gambiae s.L.,* for what? *An. coluzzii* or hybrid…etc.

Resistance mutation (L352)

Comment 7: Was the Kdr reading validated in relation to the value of the curve (CT), at the N point or by the value of FAM? What are the CT values (≤25 was positive in Kdr-E or W; 26-30 was RS-E or W) that you consider positive for the Kdr-E and the Kdr-W (RR)? For RS and SS?

- Wreting mistakes were recognized in line, L393 "Cô te d'Ivoire" and in, L394 "Bouaké ".

Discussion

Comment 1: The authors should provide information, however, moderate resistance intensity was recorded with a 10X concentration of alphacypermethrin in the Aboisso populations, and additional investigations are required for better understanding. In this study, pre-exposure to PBO showed significantly increased pyrethroid-induced mortality in all *An. gambiae s.l.* populations. However, full susceptibility was not recovered using PBO, except with the strain of Jacqueville with deltamethrin. Is there any reports document to describe this resistance in Côte d'Ivoire?

Comment 2: L449-450. The author said that the pyrrole chlorfenapyr and neonicotinoid clothianidin have new modes of action and are good candidates for malaria vector control in areas comprised of mutation and/or metabolic resistance to pyrethroids. Can you say that you can advise the authorities of your country to change the molecule or the insecticide (pyrethroids) to impregnate the mosquito nets by pyrrole chlorfenapyr and neonicotinoid clothianidin in relation to this study? Explain

Comment 3: Please, author should revise his discussion accordingly from Line 407 to 460

Conclusions

Comment 1: Lines 463-463: Check whether the revised sentence: The present study showed that *An. gambiae s.l.* was mainly composed of *An. coluzzii*, followed by *An. gambiae* s.s and hybrids in costal Côte d'Ivoire

I suggested: The present study showed that *An. gambiae s.l.* was predominate by *An. coluzzii*, followed by *An. gambiae* s.s and hybrids in the three studied sites of costal Côte d'Ivoire
