# Supplementary material for: Assessing species composition and insecticide resistance of Anopheles gambiae complex members in three coastal health districts of Côte d’Ivoire
Source: PLoS One. 2024 Dec 10;19(12):e0297604. doi: 10.1371/journal.pone.0297604 (PMC11630590; doi:10.1371/journal.pone.0297604)
Supplement: Attachment — Submitted filename: Response_to_Reviewers.docx [file pone.0297604.s004.docx]

**Response to reviewers**

**Reviewer 1**

**Comment Lines 36-37**: suggest you say “induces …” instead of “restore”

**Response :** This has been done.

**Comment Lines 45-46:** Keywords: Anopheles gambiae s.l., pyrethroids resistance, PBO, Chlorfenapyr, clothianidin, Vgsc 995F, Vgsc 995S, Ace-1 280S Coastal Côte d'Ivoire

**Response :** This has been done. See lines 46 to 47 of manuscript.

**Comment line 51**:to be removed “some of”

**Response :**This has been done

**Comment line 68** No need “burden”

**Response :**This has been done

**Comment line 78 :** Please consider revising these sentences and other as referring to the definition of species “Hybrids” individual cannot be considered as species.

**Response :** This has been done, see lines 81 to 83 of manuscript. “The species *An. gambiae s.s., An. coluzzii*, and the hybrids resulting from their interbreeding, all part of the *An. gambiae* complex, have been identified throughout the country”.

**Comment line 89-92:** Rephrase sentence from line 89 to 92 , as it may be confusing to reader. how susceptibility test can be carried out in order to have nets?

**Response :** The sentence was rephrased by : Insecticide sensitivity tests have also been conducted so that these data can facilitate the development of mosquito nets incorporating pyrethroids and other active ingredients (synergists, pyrroles, etc.) as well as combinations of active ingredients, for the upcoming LLIN distribution campaign, based on entomological stratification data. See lines 93 to 97 of manuscript.

**Comment line 97:** coma after “incidences” on line 97 and suggest change to “thousand” instead

**Response :** This has been done, see lines 104 to 105 of manuscript.

**Comment line 110:** I suggest you say “in Côte d’Ivoire” Instead

**Response :** This has been done, see line 117 of manuscript.

**Comment line 120:** should this be in present tense? In general, we use the present tense to describe actions and states of being that are still true in the present.

**Response :**This has been done, See line 126 of manuscript.

**Comment line 130-131:** There is a redundancy here with the prious sentence , considere rephrasing and/or combining them

**Response :** This has been done, see lines 136 to 137 of manuscript. The larvae and pupae were collected from larval habitats between January 2018 and December 2020.

**Comment line 132:** use “into” instead

**Response :** This has been done, see line 138 of manuscript.

**Comment line 135:** Species were identified morphologically using identification keys [26].

**Response :** The sentence has been revised and improved. See lines 141 to 142 of manuscript. Species (*An. gambiae* s.l.) were morphologically identified using identification keys [26] before being utilized for various sensitivity tests and molecular analyses.

**Comment line 138-140:** Consider combining the two sentences from lines 138 to 140, When were the tests done with the chlorfenapyr then?

**Response :** This has been done, see lines 146 to 148 of manuscript. The following tests, including intensity tests (1X, 5X, and 10X), synergist tests (PBO), and clothianidin tests, were exclusively conducted in 2020.

**Comment line 143: “**in each of the four testing tubes” instead

**Response :** This has been done, see lines 150 of manuscript.

**Comment line 148:** Please double check the PBO concentration on the coated paper, should this be 4% instead

**Response :** This has been rectified, see line 155 of manuscript.

**Comment line 155-156 :**There is need to moved thos down to line 160 before the sentence starting with “Mosquitoes…”

**Response :** This has been done, see lines 167 to 168 of manuscript.

**Comment line 165 :** Please consider revising the sentence on line 165 starting with “The tests were...” to make it clear to readers

**Response :** This has been done, see lines 173 to 174 of manuscript.

**Comment line 166 :** Is there any rational in testing against the diagnostic dose (100µg) and then 200µg (why not other concentration)

**Response :** Testing chlorfenapyr against *An. gambiae* at both the diagnostic dose (100µg) and a higher dose (200µg) serves multiple objectives. Firstly, it aids in establishing the effectiveness and potency of chlorfenapyr at the standard diagnostic concentration. Secondly, evaluating it at a higher concentration offers insights into its potential efficacy at elevated doses, particularly pertinent in scenarios of mosquito resistance or where higher concentrations are necessary for effective control.

**Comment line 166-167 :** I could not check the protocol in the given ref, so what guided the choice of these number insteaad of using 20 to 25 Mosquitoes. Why 2 days mosquitoes while it is recommend 3-5 old days moquitoes.

**Response :** The decision to use these specific numbers (15 to 20) instead of 20 to 25 mosquitoes was informed by Brogdon and Chan's 2012 protocol (https://stacks.cdc.gov/view/cdc/21777). Additionally, the age of the mosquitoes was determined based on a previous study conducted by Kouassi et al. in 2020 (DOI: 10.1186/s12936-020-03523-y).

**Comment line 168 :** Please check for the REF 29 on line 168, I am not sure if this is the good reference you should report here, as this refers to information related to epidemiology. Do add to this reference the link to make it easier to find it online.

**Response:** The accurate reference has been included, and the link is <https://stacks.cdc.gov/view/cdc/21777>.

**Comment line 171-173 :** rephrase the sentence from line 171, should be “...surviving moquitoes to exposure in WHO tubes….

**Response:** This has been done, see lines 179 to 180 of manuscript.

**General comment:** Please consider removing thoughout the manuscript, the “s.l.” or “complex members” as they indicates the same please revise all the citations in the manuscript as they do not aline with the reference listing.

**Response:** This has been done

**Comment line 176 :** Please check this, it does not seem to be correctly referenced, should it be [30]?

**Response:** This reference has been revised by [33] <https://doi.org/10.4269/ajtmh.1987.37.37>

**Comment line 176-185:** the text here seems to be copied from the manufacture guidelines/protocol. Please resentence all these and make it clear what you did instead OR to just refrered to it by citation

**Response:** The text has been rephrased, see lines 183 to 194 of the manuscript.

**Comment line 188:** please check for the right reference.

**Response:** This reference has been revised, see line 197 of the manuscript.

**Comment line 200:** please use “an” instead

**Response:** This has been done, see line 209 of the manuscript.

**Comment line 208-209:** Rephrase the sentence from lines 208 - 209, and use “master mix” instead of “mix” alone and do the same for the milar term troughout the manuscript

**Response:** This has been done, see line 226 of the manuscript.

**Comment line 217:** please check for the right reference [29].

**Response:** The reference has been updated by [37] : https://iris.who.int/handle/10665/250677

**Comment:** Is there a rational of using. I am not sure why you used ANOVA or xxx to compare proportion data here, as this a transformation before comparison. Instead I will recommend using the GLM a more modern and right way to analyse binomial data with higher power than analyses of transformed data. Since you did collection from different sites and different years I would suggest doing a GLM and assessing if MR varied between sites according to year of collection by using an interaction between site and year

**Response:** This has been done. See lines 240 to 243 and Table 3 of the manuscript. “The package ‘lsmeans’ version 2.30-0 was used for various analyses. The mortality recorded each year was compared between each insecticide (pyrethroid diagnostic concentration) and at the same site using the pairwise tests of the generalized linear model (GLM)”

**Comment line 227 :** Revising the sentence on line 227

**Response:** This has been done. See lines 247 to 248 of the manuscript.

**Comment lines 232, 233 and 340:** In the table 2 and the lines 232, 233 and 340, can you change the letters used in the formula to avoid any confusing for the readers as some of the letters (“SS”) you use there are also consider as genotypes (use for example “aa” and “AA”)

**Response:** This has been done. This has been done. See lines 252 to 253, and Table 5 of the manuscript.

**Comment line 238:** Please consider revising these sentences and other as referring to the definition of species “Hybrids” individual cannot be considered as species.

**Response:** This has been done. See lines 258 to 259 of the manuscript.

**Comment line 240:** On line 240 consider revising terms as An. gambiae ss and An. coluzzii are no longer consider as “forms” but “species”

**Response:** This has been done. See lines 260 to 261 of the manuscript.

**Comment line 243:** Revise the legend on the figure, we can really see the line on the pie charte indicating the hybrids proportions

**Response:** Figure has been replaced by Table 1

**Comment line 272:** Please be consistant in your writing, consider writing “Fig.” (example lines 247 ; 276) or “Figure” but do not mixte them

**Response:** This has been done, see line 286 of the manuscript.

**Comment lines 274 - 276:** the sentence is not very clearly explained, revise the english there

**Response :** This has been done, see lines 288 to 290 of the manuscript. In the resistance pyrethroid intensity tests, conducted on samples from Aboisso, Jacqueville, and San Pedro, mortalities remained below the 98% threshold when exposed to 1X and 5X concentrations of all insecticides.

**Comment lines 276 - 279:** Consider revising the english also the sentence from 276 to 279

**Response :** This has been done, see lines 290 to 293 of the manuscript. However, at a concentration of 10X, a high intensity of resistance was still observed across all three mosquito populations for the insecticides, except for a case of moderate intensity resistance recorded with alphacypermethrin 0.5% in the Aboisso populations, resulting in 100% mortality.

**Comment Fig 4 :** Could you indicate the number of mosquitoes exposed on the graph instead of the proportion as you did on fig 4

**Response:** This has been done, see fig 2 of the manuscript.

**Comment line 281:** Please consider using “from” instead of “of”

**Response:** This has been done, see lines 295 to 296 of the manuscript. The title has been revised by : Mortality rates of *Anopheles gambiae* s.l. population exposed to pyrethroid intensity tests from the three study populations.

**Comment 295 :** Please revise the title on line 295, Bioassays is a methods you cannot have a method within this table thus this cannot be a “bioassay”

**Response:** This has been done, see lines 309 to 310 of the manuscript. “Mortality of *An. gambiae s.l.* to pyrethroids with and without piperonyl butoxide in the study population”

**Comment lines 300 to 301:** Sentence from lines 300 to 301, please be more specific here, bars are displaying mortality from different days

**Response:** This has been done, see lines 313 to 318 of the manuscript. “After seven days of observation, *An. gambiae s.l.* populations in Jacqueville and San Pedro were found to be completely susceptible to clothianidin. The San Pedro population achieved a 100% mortality rate by the sixth day. In Jacqueville, the sensitivity threshold was reached on the seventh day, with a mortality rate of 98.81%. However, in Aboisso, susceptibility was not fully restored after seven days of post-exposure observation, with the mortality rate only reaching 55.7%.”

**Comment line 307:** Please consider removing “of” on line 308

**Response:** This has been done, see line 321 of the manuscript

**Comment line 314 :** This is a figure showing results, how can it be a “susceptibility of *An. gambiae s.l*.”

**Response:** The legend has been corrected by “Mortality of *Anopheles gambiae s.l.* exposed to 2% clothianidin in 2020 from the three study populations.” See lines 328 to 329 of the manuscript

**Comment line 318 :** Please revise the title on line 318, “CDC bottle bioassays” is a methods you cannot have a method on this figure, thus this cannot be a “CDC bottle bioassay”

**Response:** The legend has been corrected by “Mortality of *Anopheles gambiae* s.l. exposed to chlorfenapyr in CDC bottle bioassays in 2020 from the three study populations.” See lines 331 to 332 of the manuscript

**Comment line 325** : please use “was” and remove “to” instead

**Response:** This has been done, see lines 336 to 337 of the manuscript.

**Comment line Lines 328- 329:** Please consider the following “The mutation allelic frequencies were 2.3% for An. gambiae s.s. in Aboisso population and 5.7% in San Pedro population” instead.

**Response:** This has been done, see lines 340 to 341 of the manuscript. “The mutation allelic frequencies were 2.3% for *An. gambiae* s.s. in Aboisso population and 5.7% in San Pedro population”.

**Comment line 330 :** Please consider “Hybird” instead. Sentence from lines 328 - 330: Since An. gambiae ss from Aboisso has this mutation , please removed An. coluzzii and Hybrid from the brackets so that it is clear to readres that you are referring to them

**Response:** This has been done, see lines 340 to 342 of the manuscript. “The mutation allelic frequencies were 2.3% for *An. gambiae* s.s. in Aboisso population and 5.7% in San Pedro population. For the population of Aboisso, and San Pedro mutation was not detected.”

**Comment line 347-352:** Please consider revising these sentences and other as referring to the definition of species “Hybrids” individual cannot be considered as species.

**Response:** This has been done, see lines 358 to 364 of the manuscript. “The current study showed that *An. gambiae s.l.* was composed of two sibling species, namely *An. coluzzii, An. gambiae s.s*., and the product of crossbreeding. Among the two sibling species, *An. coluzzii* was the predominant species (88.24%). The samples from Jacqueville and San Pedro were composed only of *An. coluzzii*. Both sibling species were represented in the Aboisso *An. gambiae s.l.* population. Among the sibling species, *An. coluzzii* (66.95%) was most abundant, followed by *An. gambiae s.s*. and the product of hybridization.”

**Comment line 361-362:** Please consider the tense, “...was...” instead of “...has been..”

**Response:** This has been done, see line 372 of the manuscript.

**Comment line 364 - 365:** Contrasting inference here, I do not think An. coluzzii is linked to urban but the type of breeding sites as they breed preferentially in permanent water bodies such us puddles and farming sites. In addition, I could not see this from the reference [36] you cite here

**Response:** This has been done, see lines 374 to 376 of the manuscript. “*Anopheles coluzzii's* preference for hosts and specific breeding habitats, especially its attraction to the littoral region, may account for its dominance in these zones.” The reference was revised by <https://doi.org/10.1186/1472-6785-9-17>.

**Comment line 368:** Please consider revising these sentences and other as referring to the definition of species “Hybrids” individual cannot be considered as species.

**Response:** This has been done.

**Comment line 380:** Please consider revising these sentences and other as referring to the definition of species “Hybrids” individual cannot be considered as species.

**Response:** This has been done, see line 378 of the manuscript.

**Comment line 385:** Please consider using “are” instead

**Response:** This has been done, see line 395 of the manuscript.

**Comment line 403**: Pesticide is a genral term of chemical that kills pests and insecticide is a types of pesticides used against insects. Thus, please, revise the following “…including pesticides and insecticides…”

**Response:** The sentence has been revised. See lines 413 to 415 of the manuscript.

**Comment line 416:** please revise the sentence, it is not clear enough to me.

**Response:** The sentence has been revised to “The *kdr* genes are known to confer resistance to pyrethroid insecticides, which are commonly used in long-lasting insecticidal nets (LLINs) and indoor residual spraying (IRS). If the *kdr* 995F and 995S mutations spread, the effectiveness of these malaria vector control tools (LLINs and IRS) would be compromised”. See lines 428 to 431 of the manuscript.

**Comment line 446 : “**restored”

**Response:** restored was replaced by achieved. See line 461 of the manuscript.

**Comment line 453 and 456 : “**restored”

**Response:** restored was replaced by achieved. See lines 468 and 471 of the manuscript.

**Comment lines 465 - 466:** please consider “Pre-exposure to PBO...” instead

**Response:** This has been done. See line 480 of the manuscript.

**Comment lines 468 – 470** : need revision as missing the main verb and complement.

**Response:** This has been done by “*An. gambiae s.l.* proved fully susceptible to clothianidin and chlorfenapyr. This suggests that vector control tools, such as insecticide-treated nets and sprays, treated with these new insecticides could be highly effective in reducing malaria transmission in coastal Côte d'Ivoire. Their use could significantly improve malaria control and reduce the disease burden in the region”. See lines 483 to 487 of the manuscript.

**Reviewer 2**

**Comment line 82 :** move after PYR”only”

**Response :** This has been done, see line 87 of manuscript.

**Comment line 83:** Should be Strategies in your cases “tools”

**Response :** Tools has been replaced by strategies, see line 88 of manuscript.

**Comment line 127:** I suggest you used “different” instead of “diverse”

**Response :** This has been done, See line 133 of manuscript.

**Comment line 224-225** : Rephrase the sentence from line 224-225 “if corrected mortality was less than 98% at 5X diagnostic dose implied testing the 10X diagnostic dose”;

**Response:** This has been done. See lines 244 to 248 of the manuscript. “However, if corrected mortality was less than 98% at 5X diagnostic dose implied testing the 10X diagnostic dose. At 10X the diagnostic dose, a corrected mortality rate of 98-100% confirms a moderate resistance intensity. A corrected mortality rate of less than 98% at 10 X the diagnostic dose indicates a high level of resistance.”

**Comment line 245:** In some areas you seem to have high proportion of hybrids how was the mosquitoes stored (individually or in groups per tube) as the later if not managed carefully can allow sharing material such leg between speciment during the DNA extraction

**Response:** The mosquitoes are stored individually in Eppendorf tubes with silica gel, and maintained at −20 °C for subsequent identification. See lines 179 to 180 of the manuscript.

**Comment line 246 :** The stats results seem not to be alined with what is seen on the fig 3. Thus, I greatly suggest you consider redoing the analysis according to my suggestion in the data analysis section.

**Response:** We have examined the section and presented the outcomes of the diverse tests in Table 2. Furthermore, the analyses have been recalculated and are now displayed in Table 3.

**Comment line 246:** Please revise the section on “insecticide susceptibility...” there is no need to recall value for every single site at each year . It seems like a redundancy as you already present this value as a graph (Fig. 3). For example see how you did for the results on the synergist action form the PBO (from line 284)

**Response:** This has been done, see lines 267 to 278 of the manuscript. “Table 2 presents the mortality rates of *An. gambiae s.l.* populations from the surveyed sites (Aboisso, Jacqueville, and San Pedro) when exposed to pyrethroids (alphacypermethrin 0.05%, permethrin 0.75%, and deltamethrin 0.05%) over the years 2018, 2019, and 2020. Across all sites, the mortality rates for all three pyrethroids remained below 30%, confirming significant resistance of *An. gambiae s.l.* to these insecticides.

Table 3 summarizes the comparison of insecticide-induced mortality rates by site and year. In 2020, a statistically significant difference was observed in Aboisso between mortality rates due to deltamethrin and alpha-cypermethrin (estimate: 2.45; Z-ratio: 3.25; p-value=0.0033). Similarly, in 2020, permethrin also showed a statistically significant difference compared to alpha-cypermethrin in Aboisso (estimate: 3.42; Z-ratio: 3.32; p-value=0.0026). In Jacqueville, the 2020 comparison between permethrin and alpha-cypermethrin revealed a statistically significant difference (estimate: 2.08; Z ratio: 2.73; p-value=0.0174).”

**Comment lines 306 and 312:** is there any rational in using “susceptibility restored” instead of saying the “mortality rate) as “restore here may mean “going back to the original state after a lost...” and here at anytime you mentionned previous resistance to chlorfenapyr.

**Response:** This has been replaced by “induce” on line 306, see line 320 of the manuscript. And by “achieved” on line 312, see line 326 of the manuscript.

**Comment line 354: “**restored”

**Response:** restored was replaced by reestablished, see line 366 of the manuscript.

**Comment line 362:** Please consider removing “...excluding the presence of An. gambiae s.s. or hybrid...”

**Response:** This has been done.

**Comment line 399 - 401**: Do you mean, that the use of the same insecticides in bot agriculture and public health lead to target site mutations? Surprising, what causes the other resistance mechanisms then. I doubt, please make your idea clearer here.

**Response:** The argument has been replaced by **“**Phenotypic resistance to pyrethroids may indicate the presence of genetic resistance resulting from modifications to the target sites of the insecticides. This could be linked to the extensive use of the same insecticides in both agricultural and public health contexts. Additionally, other factors such as the inherent genetic variability of insect populations and agricultural practices may also contribute to the emergence and spread of resistance”. See lines 408 to 413 of the manuscript.

**Comment lines 408- 410** : there is a kind of redundancy here,, “high frequency” obviously means “high prevalence”

**Response:** The sentence has been revised to “In this study, very high allelic frequencies of the L995F *kdr* gene (>38%) were found in *An. gambiae s.l.* from the coastal health districts of Aboisso, Jacqueville, and San Pedro. These elevated allele frequencies indicate the widespread presence of the L995F and *kdr-West* mutations within the local *An. gambiae s.l.* population, as observed in several regions of Côte d'Ivoire” See lines 419 to 423 of the manuscript.

**Comment lines 417 - 419:** there is a kind of redundancy here,, “high frequency” obviously means “high prevalence”

**Response:** The sentence has been revised to “The high frequencies of the G119S mutation in Aboisso and Jacqueville suggest its widespread presence in the coastal areas of Côte d'Ivoire.” See lines 431 to 433 of the manuscript.

**Comment lines 421 - 423:** Please consider revisiting lines 421 - 423

**Response:** The sentence has been revised to “The present study demonstrates that increased insecticide concentrations and PBO pre-exposure significantly heightened mortality in all three coastal populations of An. gambiae s.l. against all pyrethroids. However, this approach did not fully achieve susceptibility. See lines 435 to 437 of the manuscript.

**Comment lines** 427 - 428: the sentence is not clear enough, how could a “reduced insectidal activity” be associated with “lower personal protection”

**Response:** The sentence has been revised to “Reduced insecticidal activity can result in lower personal protection by allowing more insects to survive. This increases the likelihood of bites and the spread of insect-borne diseases, thereby threatening the effectiveness of LLINs.” See lines 441 to 443 of the manuscript.

**Comment lines 428 and 430:** Please consider revising the citation [29] as i suggested earlier

**Response:** The citation has been revised to doi:10.1186/s12936-019-2685-2

**Comment lines 436 - 438:** Please consider revisiting lines 421 - 423 and lines 436 - 438 as they seem to bring the same information.

**Response :** The sentence has been revised by “Although insecticide-induced mortality increased, full sensitivity with PBO was only observed in the Jacqueville strain with deltamethrin”. See lines 451 to 453 of the manuscript.

**Comment lines 448-449:** please revise the term “...pyrrole chlorfenapyr...neonicotinoid clothianidin”

**Response:** This has been revised by “chlorfenapyr (pyrrole) and clothianidin (neonicotinoid)”. See lines 463 to 464 of the manuscript.

**Reviewer 3**

**Comments 1:** Line 1 and 2. It’s will be important to reformulate the title of the manuscript I’m suggested: Assessing of species composition and insecticide resistance of *Anopheles gambiae* complex member in three coastal health districts of Côte d’Ivoire

**Response 1:** This has been done, see lines 1 to 2 of manuscript “Assessing species composition and insecticide resistance of *Anopheles gambiae* complex member in three coastal health districts of Côte d’Ivoire”

a. **Abstract:**

**Comment 1:** Line 33: Author should put the 4.7% of hybrid species for what species?

**Response 1:** The sentence has been revised and improved, see line 32 of manuscript. The 4.17% were put for the crossbreed (*An. coluzzii/An. gambiae* *s.l.*)

**Comment 2**: Author should put the abbreviation (An. gambiae s.L) throughout the abstract subsections as the species name “Anopheles gambiae sensu lato” was already mentioned at the beginning of the abstract in line 41.

**Response 2:** Species abbreviations have been included in all sub-sections of manuscript.

**Comment 3**: Abstract and throughout the manuscript, common Latin terms such as “s.s” and “s.L” are not italicized

**Response 3 :** This has been done

**Comment 4:** The authors are not mentioned in the abstract and the methodology if this is a cross-sectional survey, a longitudinal survey…etc. Add in the abstract and the methodology (Study site)

**Response  4:**The type of study has been added. See line 24 of manuscript.

b. **Introduction:**

**Ccomment 1:** This is a study of the An. gambiae complex, except that I have only seen two species of the complex mentioned in the manuscript. The authors needs to define the An. gambiae complex and to cite the nine member of An. gambiae s. L. These articles will help you to cite the different species of the An. gambiae complex member, those which contribute to transmission and those which do not contribute. Loughlin SO (2020) <https://doi.org/10.1080/20477724.2020.1722434>. Abel et al. (2024) <https://doi.org/10.1186/s13071-023-06102-7>

**Response 1:** Information has been added. See lines 78 to 80 of manuscript.

**Comment 2**: The authors are not mentioned the Entomological Inoculation Rate (EIR) of An. gambiae s.L in coastal Côte d'Ivoire (in the studied sites)

**Response 2 :** Information has been added, see line 102 of manuscript. “Although a low entomological inoculation rate (10.9 ib/p/y in Bardo) is recorded in the coastal zone of Côte d'Ivoire, this area has reported a high incidence of malaria.”

c. **Methodology:**

**Comment 1:** Line 126 and 135. The larval method collected is not mentioned for what? What do you collected in the water wells, natural breeding sites, gardens field …etc larval only or not?

**Response 1:** The larvae collection method has been added. See lines 133 to 137 of manuscript.

**Comment 2 :** For rearing adult, author need to mention the technics used of rearing adult mosquito and the water type

**Response 2:** The technics used of rearing adult mosquito and the water type has been added.

**Comment 3:** Line 136: WHO tube bioassay assesses the degree of susceptibility for what?

**Response 3:** The WHO tube bioassay evaluates the sensitivity of adult female *An. gambiae* s.l. aged 2 to 5 days to insecticides, including 1X, 5X, and 10X diagnostic doses of pyrethroids (alphacypermethrin, deltamethrin, and permethrin), as well as a combination of PBO synergists with a 1X diagnostic dose of pyrethroids, and clothianidin.

**Comment 4** : Line 137 to 139: The author mentioned that the diagnostic dose tests of pyrethroids (alphacypermethrin, deltamethrin, and permethrin) were conducted in 2018, 2019, and 2020. The following tests were conducted exclusively in 2020 on the same population of An. gambiae complex (Fo, F1, F2…)?

**Response 4:** All tests were conducted on Fo from the same population of *An. gambiae* *s.l.,* including those in 2020. See line 149 of manuscript.

**Comment 5:** The bottle analysis method is a monitoring tool for detecting and characterizing changes in insecticide susceptibility in vector populations?

**Response 5:** The bottle analysis method enables the early detection and characterization of changes in insecticide susceptibility within vector populations. This technique entails exposing mosquitoes to precise doses of insecticides contained within bottles, thereby assessing their reaction and establishing their resistance level.

**Comment 6**: Mosquito sample processing for DNA extraction was not mentioned clearly, does the author used whole mosquito for extraction or it was dissected to head and thorax and abdomen?

**Response 6 :** DNA extraction was performed using a whole mosquito. See lines 179 to 180 of manuscript.

**Comment 7**: L202: For molecular Identification of target site mutation, author need to mention the primers used and PCR probes sequences and brief about the PCR methods.

**Response 7:** Information has been added. See lines 214 to 231 of manuscript. The primers kdr-Forward (5'-CATTTTTCTTGGCCACTGTAGTGAT-3') and kdr-Reverse (5'-CGATCTTGGTCCATGTTAATTTGCA-3') were standard oligonucleotides without any modifications. The probe WT (5'-CTTACGACTAAATTTC-3'), labeled with HEX at the 5' end, was used for detecting the wildtype allele. The probes kdr W (5'-ACGACAAAATTTC-3') and kdr E (5'-ACGACTGAATTTC-3') were labeled with FAM for detecting the kdr-w and kdr-e alleles, respectively. Similarly, the primers Ace1-Forward (5'-GGCCGTCATGCTGTGGAT-3') and Ace1-Reverse (5'-GCGGTGCCGGAGTAGA-3') were also standard oligonucleotides. The probe WT (5'-TTCGGCGGCGGCT-3') was labeled with HEX, while the probe (5'-TTCGGCGGCAGCT-3') was labeled with FAM.

**Comment 8:** L205-205: The author said that the krd (Kdr-East and Kdr-West) mutation was investigated using real-time PCR (TaqMan assay). To my knowledge, the probes (Kdr-East and West) attach to the FAM, I need to know how do you differentiate it? Did you do two separate the mix-PCR (WT, sensimix, water and Kdr-E FAM; WT, sensimix, water and Kdr-W FAM) or in the same mix did you put the Kdr-E and Kdr-W?

**Response 8:** During this study, the master mixes were prepared separately for each mechanism studied.

**Comment 9:** Please explain how this statistical analysis plan answer the stated question,: role of individual members of An. gambaie compex to malaria transmission?

**Response 9:** The statistical analysis plan assesses the role of individual members of the *An. gambiae* complex in malaria transmission by determining the resistance status of each mosquito population according to WHO criteria. It adjusts for mortality and compares mortality annually between each insecticide. It also evaluates resistance intensity and the effects of synergists, while determining the frequency of resistance mutations.

**Comment 10:** Were there any considerations for possible non-blood-fed females from exophagic, zoophagic and endophilic mosquitoes? Any chance that some mosquitoes feed?

**Response 10:** The bioassay performed according to the WHO protocol involved exposure of 2–5-day old nonblood-fed female adults. See lines 150 and 1175 of manuscript.

d. **Results:**

**Comment 1, L236:** For molecular identification of Anopheles gambiae complex member species, author mentioned the SINE200 or SINE-PCR using (Line 187 to 188). To my knowledge, SINE PCR makes it possible to identify three species of the *An. gambiae* complex, namely: *An. gambiae ss, An. arabesnsis* and *An. coluzzii*. This is what I don't see in this manuscripts, in these results, the author identify two species and the hybride, were these others species in the complex member?

**Response 1:** The hybrids identified here do not belong to the *An. gambiae* complex and are not distinct species. Instead, they are the result of crosses between the two species *An. coluzzii* and *An. gambiae* s.s.

**Comment 2: L237:** the author said that 672 *An. gambiae* s.l*.* was selected from the three studied sites for species identification, however only 671 samples were used in *An. gambaie s.l* species molecular identification (there is 1 sample undetectable), because when I do the calculations of 593+51+27=671, where is the remaining 1 mosquito?

**Response 2:** This was a data entry error; the number of hybrids was 28, not 27. Therefore, the sum will be 593 + 51 + 28 = 671, not 593 + 51 + 27 = 671.

**Comment 3:** Figure 2 is not adopted for these results, remove that. It is better to represent the distribution of *An. gambiae s.l.* species on a table

**Response 3:** This has been done. The figure has been replaced by Table 1

**Comment 4: L244**, Remove the “of members” and write in the Table XX: Distribution of *Anopheles gambiae s.l.* in the three studied site

**Response 4:** This has been done

**Insecticide susceptibility in *An. gambiae s.l***

**Comment 5:** How many Anopheles have been testing with each insecticide, years and the different studied sites? The number of each *An. gambiae s.L*. alive and death (e.g: 2% (20/672))?

**Response 5:** Number of dead mosquitoes tested for each insecticide applied (Deaths/Total number exposed)

**Intensity of resistance section**

**Comment 6, L273-273:** The author said that: these results confirmed the strong resistance of *An. gambiae s.L.,* for what? *An. coluzzii or hybrid*…etc.

**Response 6:** The results confirm the high resistance of *An. gambiae* s.l. as the mosquitoes used in the various bioassays were only identified morphologically. It should be noted that genotypic characterization was conducted after the bioassays. Hence, species within the *An. gambiae* complex was not specified. Furthermore, only a few samples were utilized for the genotypic characterization of *An. gambiae* s.l.

**Resistance mutation (L352)**

**Comment 7:** Was the Kdr reading validated in relation to the value of the curve (CT), at the N point or by the value of FAM? What are the CT values (≤25 was positive in Kdr-E or W; 26-30 was RS-E or W) that you consider positive for the Kdr-E and the Kdr-W (RR)? For RS and SS?

- Wreting mistakes were recognized in line, L393 “Côte d'Ivoire” and in, L394 “Bouaké ”.

**Response 7:** The Kdr reading was validated against the FAM value. See lines 229 to 230 of manuscript. In our study, the control CT values deemed positive for RR, RS, and SS of Kdr-E, Kdr-W and Ace-1 were as follows, respectively:

| **Site cible** | **Statut** | **FAM Ct values** | **HEX Ct values** | **Mean Ct values** |
| --- | --- | --- | --- | --- |
| Kdr East | RR | 20 | 22,87 | 21,43 |
| Kdr East | RS | 24,9 | 27,3 | 26,1 |
| Kdr East | SS | 23,12 | 24,75 | 23,93 |
| Kdr West | RR | 23,3 | 25,01 | 24,15 |
| Kdr West | RS | 33,12 | 33,79 | 33,45 |
| Kdr West | SS | 27,12 | 22,79 | 24,95 |
| Ace_1 | RR | 22,65 | 24,13 | 23,39 |
| Ace_1 | RS | 28,85 | 28,33 | 28,59 |
| Ace_1 | SS | 24,28 | 22,77 | 23,52 |

**-** These are not spelling mistakes: ‘Côte d'Ivoire’ is the name of the country and ‘Bouaké’ the name of the town.

**Discussion**

**Comment 1:** The authors should provide information, however, moderate resistance intensity was recorded with a 10X concentration of alphacypermethrin in the Aboisso populations, and additional investigations are required for better understanding. In this study, pre-exposure to PBO showed significantly increased pyrethroid-induced mortality in all *An. gambiae s.l*. populations. However, full susceptibility was not recovered using PBO, except with the strain of Jacqueville with deltamethrin. Is there any reports document to describe this resistance in Côte d’Ivoire?

**Response 1 :** The moderate resistance intensity recorded to alphacypermethrin in the Aboisso populations marks the first occurrence in Côte d'Ivoire. However, Kouassi et al. (2020) https://doi.org/10.1186/s12936-020-03523-y noted moderate resistance against permethrin in Nassian and Odienné. As for the complete sensitivity restored with PBO for the Jacqueville strain with deltamethrin, this represents the first case in Côte d'Ivoire.

**Comment 2:** L449-450. The author said that the pyrrole chlorfenapyr and neonicotinoid clothianidin have new modes of action and are good candidates for malaria vector control in areas comprised of mutation and/or metabolic resistance to pyrethroids. Can you say that you can advise the authorities of your country to change the molecule or the insecticide (pyrethroids) to impregnate the mosquito nets by pyrrole chlorfenapyr and neonicotinoid clothianidin in relation to this study? Explain

**Response 2 :** Based on this study, I would advise the authorities of our country to consider seriously replacing the pyrethroids traditionally used to impregnate mosquito nets with the chlorfenapyr (pyrrole) and the clothianidin (neonicotinoid). The study findings indicate better mosquito mortality when these two substances are used compared to pyrethroids. This transition could potentially enhance the effectiveness of mosquito nets in combating disease vectors such as malaria, thus providing increased protection to the population. A more thorough analysis of the benefits and practical implications of this change, in consultation with experts and stakeholders, would be necessary to develop an effective implementation strategy.

**Comment 3:** Please, author should revise his discussion accordingly from Line 407 to 460

**Response 3 :** This has been done

**Conclusions**

**Comment 1:** Lines 463-463: Check whether the revised sentence: The present study showed that *An. gambiae s.l.* was mainly composed of *An. coluzzii*, followed by *An. gambiae s.s* and hybrids in costal Côte d’Ivoire

I suggested: The present study showed that *An. gambiae s.l.* was predominate by *An. coluzzii,* followed by *An. gambiae s.s* and hybrids in the three studied sites of costal Côte d’Ivoire

**Response 1 :** This has been done. See lines 276 to 278 of manuscript.
