# Supplementary material for: Assessing species composition and insecticide resistance of Anopheles gambiae complex members in three coastal health districts of Côte d’Ivoire
Source: PLoS One. 2024 Dec 10;19(12):e0297604. doi: 10.1371/journal.pone.0297604 (PMC11630590; doi:10.1371/journal.pone.0297604)
Supplement: Attachment — Submitted filename: Response_to_Reviewers.docx [file pone.0297604.s005.docx]

**Response to reviewers**

**Reviewer 3**

**Comment 1:** For the An. 32 gambiae s.l. populations were primarily composed of Anopheles coluzzii (88.24%, n = 312), followed by Anopheles gambiae sensu stricto (7.56%) and the crossbreed (4.17%), I am suggested that: In the total of 672 *An. gambiae* S.L. analysed, 27 (4.17%) mosquitoes were not discriminated against; thus, they are part of other species of *An. gambiae* complex.

**Response 1:** We thank the Reviewer #3 for drawing our attention on hybrids identified as member of *An gambiae* *s.l.* complex identified in our present study. Please, note that the 27 mosquitoes identified as hybrids are genuine hybrids and not undetermined species. Their identification was based on the Santolamazza et al. method, which has been validated in prior research. For instance, Fassinou et al. (2019, Trop Med Health, 11:47:23, DOI: [10.1186/ s41182-019-0151-z](https://doi.org/10.1186/s41182-019-0151-z)) explicitly described the use of the S200 X6.1 primer set to identify the hybrid form (M/S) between *An. coluzzii* and *An. gambiae* s.s., as indicated in the legend of Figure 2 (page 5) in their publication. Furthermore, Kacou et aL (2024, Med Vet Entomol, 2024; DOI: 10.1111/mve.12759), in their recent research conducted in Côte d’Ivoire, confirmed the identification of hybrids (M/S) using the same S200 X6.1 primer set method. Additionally, the identification of hybrids in Coast Côte d’Ivoire is novel discovery. Indeed, Caputo et al. (2020, Infect Genet Evol, 98:105215; DOI: 10.1016/j.meegid.2022.105215) identified the presence of *An. coluzzii*-*An. gambiae* s.s. hybrids in Coast and inland Anopheles gamabiae s.l. complex in Côte d’Ivoire. Therefore, based on these established approaches and previous findings in similar sites in Côte d’Ivoire, we confirm that the 27 mosquitoes identified in our study are indeed *An. coluzzii*-*An. gambiae* s.s hybrids within the *An. gambiae* complex.

**Comment 2: If the hybrids identified here do not belong to the *An. gambiae* complex and are not distinct species, why do you identify those in An. gambiae complex if you used PCR? I think your work is based on the member An. gambiae? Replace the hybrids with: other species of *An. gambiae* complex Because this complex are nine species**

**Response 2:** Thank you very much for your thoughtful comments and suggestions. Please as indicated above, we would like to clarify that all mosquitoes used in this study were morphologically identified as *An. gambiae* *s.l.* prior to molecular analysis. Moreover, to date, the only subspecies of the *Anopheles gambiae* complex identified in the district under study are *An. coluzzii* and *An. gambiae s.s.,* along with hybrids of these two subspecies, as shown in previous studies by Tondossama et al. (2023; DOI: [https://doi.org/10.3390/insects 14090758](https://doi.org/10.3390/insects%2014090758)), Yokoly et al. (2021; DOI: <https://doi.org/10.1186/s12936-021-03938-1>) and Caputo et al. (2020; DOI: 10.1016/j. meegid.2022.105215). Therefore, the individuals encountered in our study could only be the result of crossbreeding between *An. coluzzii* (M) and *An. gambiae* s.s (S), making the term "hybrids (M/S)" the most appropriate. These hybrids are indeed part of the *Anopheles gambiae* complex, as confirmed by our PCR analysis. The two other Reviewers. We believe it is unnecessary to replace "hybrids" with "other species of the *An. gambiae* complex," as these individuals are hybrids from two subspecies (i.e. *An. coluzzii* (M) and *An. gambiae* s.s (S)) within the complex *An. gambiae s.l.* Therefore, no requested change has been made to our revised manuscript R1.

We hope this clarification addresses your concerns.
